# High Energy Density in Azobenzene-based Materials for Photo-Thermal Batteries via Controlled Polymer Architecture and Polymer-Solvent Interactions

**DOI:** 10.1038/s41598-017-17906-w

**Published:** 2017-12-19

**Authors:** Seung Pyo Jeong, Lawrence A. Renna, Connor J. Boyle, Hyunwook S. Kwak, Edward Harder, Wolfgang Damm, Dhandapani Venkataraman

**Affiliations:** 1 Department of Chemistry, University of Massachusetts Amherst, 710 North Pleasant Street, Amherst, Massachusetts 01003-9303 USA; 2 Schrödinger, Inc., Cambridge, MA 02142 USA; 3 Schrödinger, Inc., New York, NY 10036 USA

## Abstract

Energy densities of ~510 J/g (max: 698 J/g) have been achieved in azobenzene-based syndiotactic-rich poly(methacrylate) polymers. The processing solvent and polymer-solvent interactions are important to achieve morphologically optimal structures for high-energy density materials. This work shows that morphological changes of solid-state syndiotactic polymers, driven by different solvent processings play an important role in controlling the activation energy of *Z*-*E* isomerization as well as the shape of the DSC exotherm. Thus, this study shows the crucial role of processing solvents and thin film structure in achieving higher energy densities.

## Introduction

Azobenzene units with appropriate molecular packing are excellent candidates for active layers in photo-thermal batteries (PTBs) and the predicted energy densities are higher than the energy densities in current electrochemical batteries^1–5^. Guided by these predictions, azobenzene units have been anchored on scaffolds such as carbon nanotubes and reduced graphene oxide (rGO), achieving energy densities approaching ~490 J/g^2,4^. Yet, placing azobenzene units at precise locations on these scaffolds is synthetically challenging. We hypothesize that controlled polymerization techniques can provide convenient synthetic pathways to organize azobenzene units on polymer scaffolds with the requisite molecular packing for high energy densities. Herein, we show that poly(methacrylate) with pendant azobenzene units, poly(4-phenylazophenyl methacrylate) (AzoPMA), can have energy densities up to 698 J/g, with an average energy density of 510 ± 115 J/g. We demonstrate the critical role of polymer-solvent interactions on (1) the assembly of AzoPMA to form morphologies for high energy density storage and (2) the shape of DSC exotherm. Lastly, the processing solvent plays an important role in tuning the isomerization kinetics of the AzoPMA.

In organic PTBs, light converts a stable isomer into a high-energy meta-stable isomer (charging). The meta-stable isomer can be converted back to the low-energy isomer on-demand (discharging)^6–8^, where the excess energy (energy difference: *∆H*) is typically released as heat. Since this configurational isomerization does not generate by-products, the charging-discharging cycle can be repeated without loss of active material. Azobenzene-based molecules have emerged as strong candidates for PTBs because of their ease of synthesis, high quantum yield of *E-Z* photoisomerization, high absorption cross-section in UV-Vis light, and ease of chromophore tunability for light absorption and kinetics of isomerization^9–11^. However, until recently, the major drawback of these systems has been their relatively low-energy densities^12^.

Computational studies by Kolpak *et al*. predict that azobenzene units placed 4.24 Å apart on a rigid scaffold, such as carbon nanotubes (CNTs), can lead to dramatic increases in energy storage densities—from ~200 J/g for unassembled azobenzene units to 820 J/g for azobenzene molecules assembled on CNTs^1^. For comparison, a lithium-ion battery has an energy density between 400 J/g to 650 J/g^13^. Recent experimental studies by Kucharski *et al*. showed that energy densities of ~217 J/g can be obtained in azobenzene units attached to CNTs; the relatively low-energy density was attributed to inefficient grafting of azobenzene units to CNTs^2^. Other experimental studies show that azobenzene molecules anchored on rGO can lead to energy densities of ~490 J/g^4^.

We hypothesized that we can achieve high energy densities by (a) anchoring azobenzene on polymer backbones to achieve high functional group density in a polymer chain, (b) placing end-groups on the polymers that can self-assemble into cylindrical structures, similar to CNTs, and (c) tuning polymer-polymer interaction by choosing different processing solvents. To test our hypotheses, we chose poly(methacrylate) (PMA) as our backbone, which is known to have a preference for high syndiotacticity in radical polymerizations^14–16^. In PMA, the distance between two syn ester groups in a syndiotactic triad is calculated to be ~5 Å, similar to distance between azobenzene units on CNTs. We chose hexabenzocoronene (HBC) as our end-group because it can self-assemble through π-π stacking interactions into cylindrical structures^17,18^. In addition, we tested two reference solvents with low boiling point and high solubility for the polymer, dichloromethane (DCM) and tetrahydrofuran (THF), to understand role of polymer-solvent interaction and optimal structure for high energy density.

## Results and Discussion

### Synthesis and Characterization of AzoPMAs

**Figure 1:**
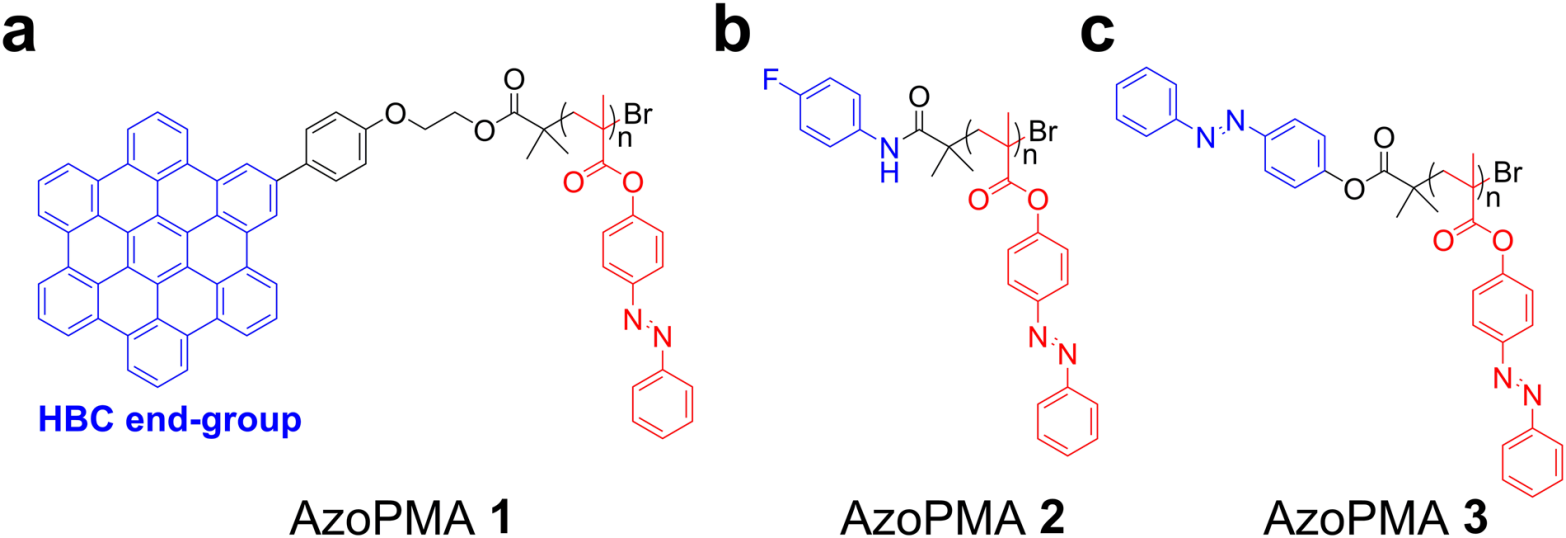
Chemical structures of AzoPMA polymers with different end-groups. AzoPMA polymers were synthesized by SARA ATRP. Polymers were synthesized with (**a**) a HBC end group for π-π stacking interactions between polymer chains (AzoPMA **1**), (**b**) a 4-fluorobenzamido end group for hydrogen bonding interactions (AzoPMA **2**), or (**c**) a 4-phenydiazenyl-benzoate end group as a control (AzoPMA **3**).

The PMA-based polymer bearing pendant azobenzene units and HBC as the end group (AzoPMA **1**) was synthesized using a previously reported supplemental activator and reducing agent atom transfer radical polymerization (SARA ATRP)^19^. As a control, we synthesized PMA with 4-fluorobenzamido (AzoPMA **2**) and 4-phenydiazenyl-benzoate end-groups (AzoPMA **3**), see Fig. 1. (Supplementary Fig. S1–14) Polymer tacticity estimates from ^13^C NMR showed that AzoPMA **3** is syndiotactic-rich, as expected from SARA ATRP, with 73.6% of rr (Supplementary Fig. S15). Thermogravimetric analysis (TGA) of these polymers (Supplementary Fig. S20) showed no mass loss until 200 °C, which indicated that the polymers are thermally stable in the temperature range of 0 °C–140 °C for differential scanning calorimetry (DSC) studies to evaluate the energy density.

### Energy Density of Syndiotactic AzoPMAs

**Figure 2:**
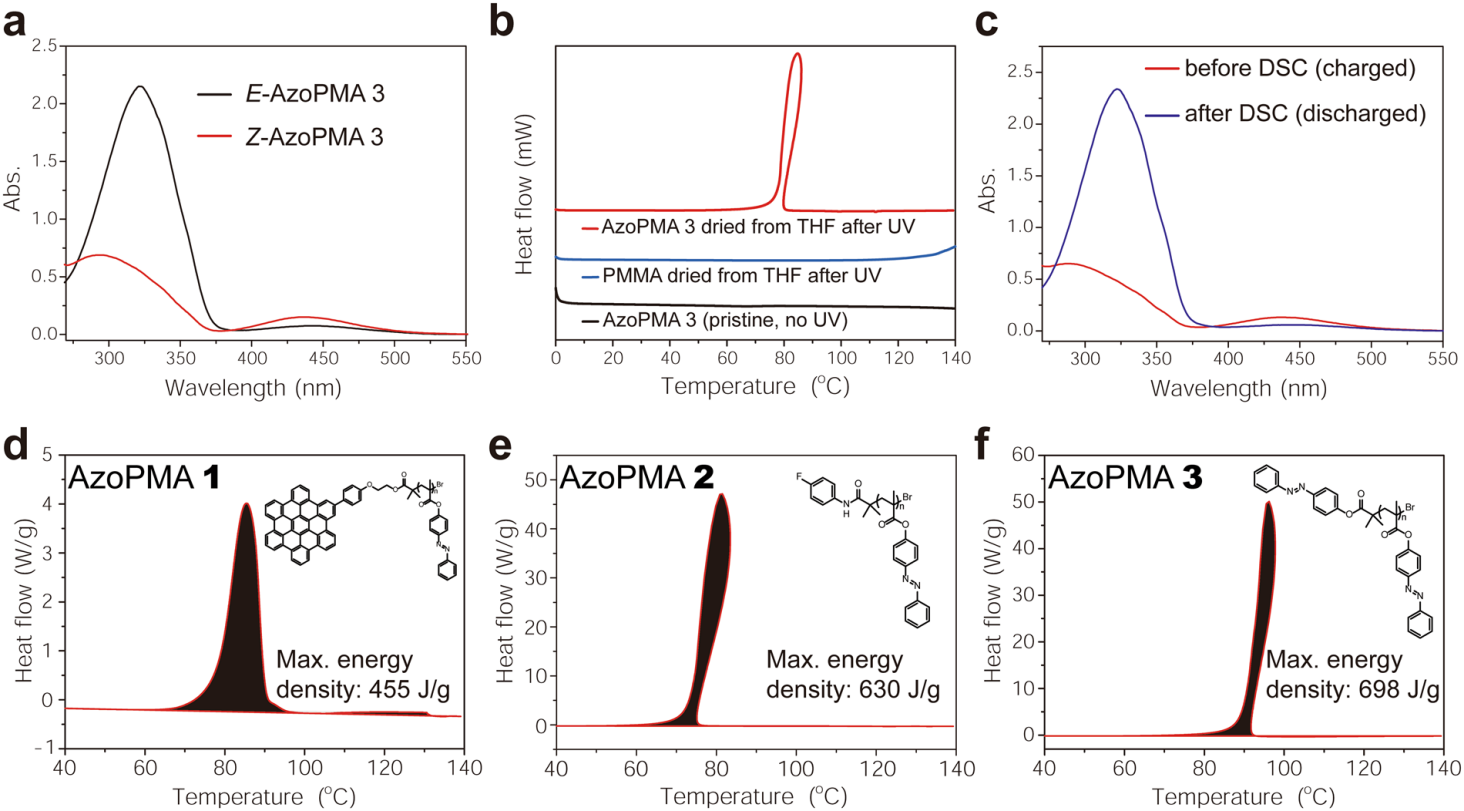
(**a**) UV-Vis of *E* and *Z*-AzoPMA **3**. (**b**) DSC curves of pristine AzoPMA **3**, AzoPMA **3** dried from THF after UV, and PMMA dried from THF after UV in the 1st heating. (**c**) UV-Vis of AzoPMA **3** before/after DSC measurement. (**d**–**f**) Maximum energy density DSC curves for AzoPMAs. AzoPMA polymers show an exothermic peak on their first heating cycle at 60 °C–100 °C, which is indicative of the isomerization of the *Z*-isomer to the *E*-isomer during discharging. The maximum exotherm for (**d**) AzoPMA **1** has an energy density of 455 J/g. For (**e**) AzoPMA **2**, the maximum energy density was 630 J/g. The highest energy density was obtained from **(f**) AzoPMA **3**, at 698 J/g.

To measure the energy density of the syndiotactic-rich AzoPMAs, samples were prepared by dissolving the polymers in THF for DSC analysis because it is difficult for azobenzene materials to isomerize from *E*- to *Z*-isomer in the solid state (Supplementary Fig. S32). Then, the solution was irradiated with UV radiation (~365 nm) to photoisomerize the *E*-isomer to the *Z*-isomer (‘charging’) (Fig. 2a). The flask containing the solution was then wrapped with aluminum foil and the solvent was removed in vacuo to get a solid sample. This solid sample was dried in vacuo for 5 h in the dark to remove any residual solvent. The samples were then hermetically sealed in aluminum pans for DSC analysis. All samples were subjected to a heating-cooling cycle followed by a second heating cycle. In the first cycle, the temperature was raised from 0 °C to 140 °C, and then cooled back to 10 °C at the rate of 5 °C/min. In the second heating, the temperature was raised from 10 °C to 150 °C at the rate of 5 °C/min. All polymers showed a single exotherm around 60 °C–100 °C in the first heating cycle. Except for this exotherm, the DSC traces were featureless (Supplementary Fig. S21). This single exotherm was not observed in the second heating of DSC and in polymers not exposed to UV radiation. This exotherm was also not observed in the UV-irradiated poly(methyl methacrylate) (PMMA) dried from THF (Fig. 2b). After discharging, we checked the discharged DSC sample by UV-Vis, and all *Z*-isomers were converted back to its original stable *E*-isomer (Fig. 2c).

For AzoPMA **1**, the energy density was found to be 421 ± 48 J/g, comparable to the energy density obtained for azobenzene anchored on rGO^3,4^. We expected that AzoPMA **2** and **3** will have lower energies compared to AzoPMA **1** because the end groups on these polymers are not expected to facilitate self-assembly through π-π stacking interactions. Contrary to our expectation, we obtained average energy densities of 444 ± 107 J/g for AzoPMA **2** and 510 ± 115 J/g for AzoPMA **3**. The maximum energy density of 698 J/g was observed for AzoPMA **3**. These numbers indicated that the end-group was unnecessary and possibly even detrimental to achieving high-energy density values. The exotherm observed from AzoPMA **3** was sharp, which indicates uniformity in the azobenzene environment and/or a cooperative isomerization of the pendent units^20^. We also observed that some of the exotherms had an anomalous peak shape, which we found was because of the non-monotonic character of the temperature during the measurement, due to the large burst of heat from the sample (Supplementary Fig. S22). The area under the exotherm was used to calculate the energy density of the polymers. Maximum DSC curves are for AzoPMA **1**–**3** are shown in Fig. 2d–f respectively. To the best our knowledge, the energy density of AzoPMA **3** dried from THF (698 J/g) is the highest for azobenzene-based systems^13,21^. We found that the charging-discharging cycled can be recycled without loss of energy density (Supplementary Fig. S33).

To account for the observed energy density of the AzoPMA **3** fabricated from THF, we probed for the possible presence or incorporation of inadvertent impurities in the polymer materials. We first considered the possibility of THF photo-degradation products generated during the charging process using UV light contributing to the energy density. We examined this possibility by irradiating a solution of AzoPMA **3** in DCM with UV radiation to convert the *E*-isomer to the *Z*-isomer. The DCM solvent was removed under vacuum, and the polymer was re-dissolved in THF. The sample was prepared for DSC measurement as described above and it showed a maximum energy density of ~540 J/g (Supplementary Fig. S23). Moreover, there is no exotherm found in the UV-irradiated poly(methyl methacrylate) (PMMA) dried from THF (Fig. 2b). These experiments ruled out the possibility that the exotherm observed in DSC is from THF photo-degradation products. We then considered the possibility of the presence of residual reagents from the SARA ATRP. Energy dispersive analysis did not show any peaks attributable to copper and ^1^H NMR did not show any peaks attributable to the amine (Me_6_TREN), which was used as a ligand (Supplementary Fig. S25). From all of these experiments, we concluded that the observed exotherm in *Z*-AzoPMA **3** is associated with discharging, is exclusively from the isomerization of the *Z*-isomer to the *E*-isomer, and is consistent with observations made by others^2,3,22–24^. We thus used AzoPMA **3** for the remainder of our studies.

### Solvent Effect

The control experiments indicated that the samples obtained from DCM have substantially lower energy densities than samples obtained from THF. We reasoned that solvents can change the packing density and morphology of a polymer^25–27^. Solvents can also alter the kinetics of isomerization of azobenzene molecules^28–32^. To understand the role of solvent on the energy densities of AzoPMA **3**, we chose to use THF and DCM as reference solvents among various organic solvents. THF and DCM are excellent solvents for AzoPMA **3**. Polymer chains extend in a good solvent which provides an environment for high polymer-polymer interactions^27^. They have low boiling points which allow their removal at low temperatures without adversely impacting the *Z*/*E* isomer ratio. They also have similar dipole moments (1.6 D for THF to 1.75 D for DCM) and similar viscosities (0.48 cP for THF and 0.41 cP for DCM), and thus their impact on the kinetics of isomerization should be similar. A key difference between these two solvents is the nature of the polymer-solvent interaction. The polymer-solvent interaction parameter (*χ*) is −0.15 to 0.09 for poly(methylmethacrylate) (PMMA)-DCM and *χ* = 0.44 to 0.46 for PMMA-THF, indicating that the PMA backbone may have a stronger interaction with DCM than with THF^33^. Thus, using THF and DCM allows us to elucidate the role of the polymer-solvent interaction on the observed differences in energy density.

**Figure 3:**
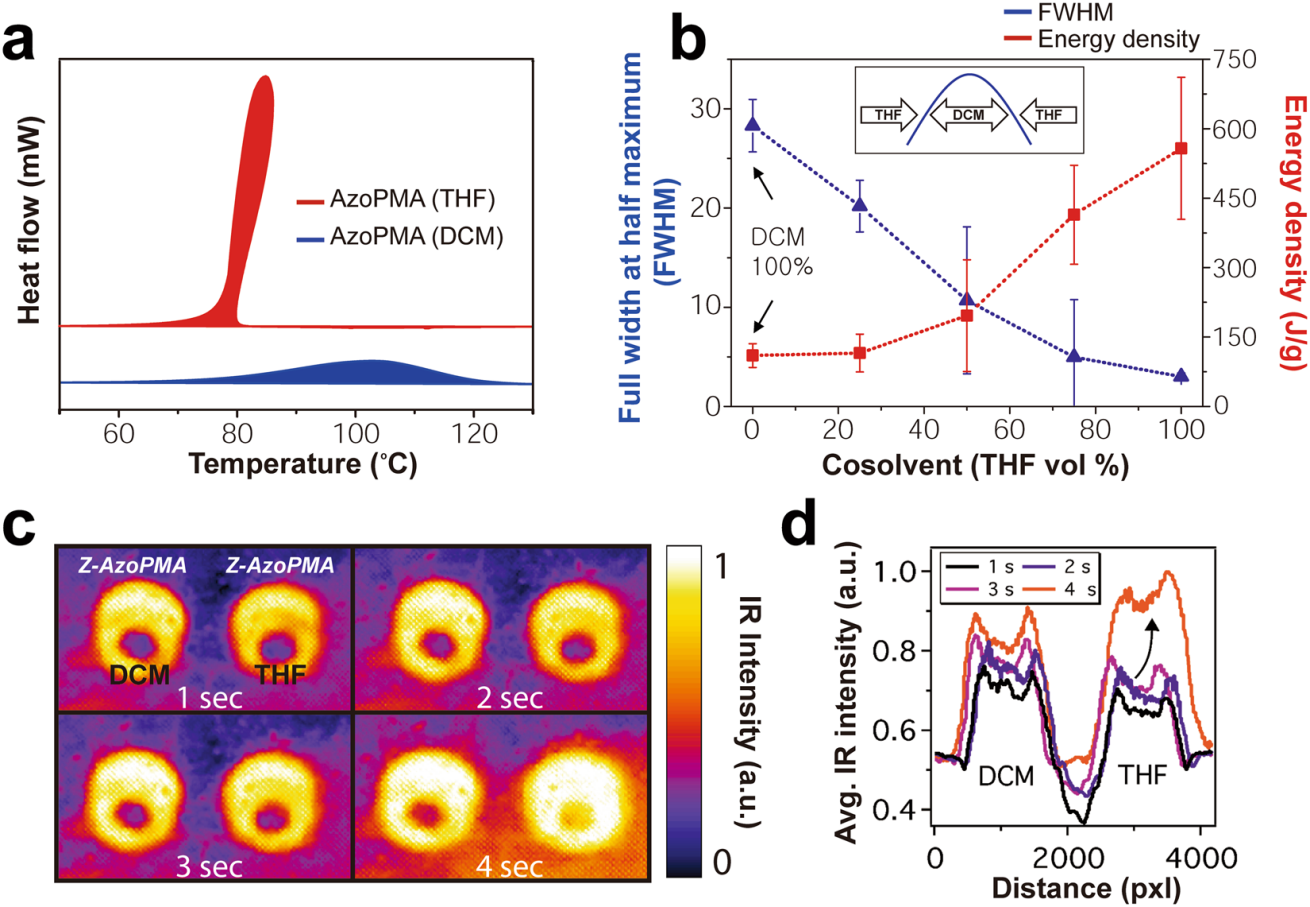
(**a**) DSC exotherms, during the first heating cycle, from the same *Z*-AzoPMA **3** dried from either THF or DCM. AzoPMA **3** dried from THF showed sharp exotherms with high-energy densities. Conversely, AzoPMA **3** dried from DCM showed a broad exotherm with relatively low energy densities. (**b**) Diagram of FWHM and energy density of AzoPMA **3** based on the different co-solvent ratios of THF and DCM. FWHM value (unit: °C, temperature difference) was calculated from the width of the DSC exotherm curve. Inset is a representation of DSC curve change based on different solvent ratio. (**c**) IR imaging of hermetically sealed aluminum DSC pans containing *Z*-AzoPMA **3** dried from THF or DCM. The DSC pans were place on a hot plate (>100 °C) and monitored with an IR camera. Each frame (from top left) represents the IR image after approximately one elapsed second, where the left pan is AzoPMA **3** dried from DCM, and the right dried from THF. (**d**) A plot of average IR intensity as a function of distance across the frame shows the average IR intensity profiles of the AzoPMA **3** samples over time.

We prepared DSC samples of AzoPMA **3** as before, except either THF or DCM was used as the initial (‘charging’) solvent. For samples obtained from DCM, AzoPMA **3** consistently displayed lower energy densities compared to solid samples obtained from THF (see Fig. 3a). Samples dried from DCM had an average energy density of 110 ± 25 J/g compared to 510 ± 115 J/g from THF (more than three trials). More importantly, the DSC traces of samples obtained from DCM samples were broad whereas the DSC traces from THF samples were narrow. We then mixed THF with DCM in various ratios and measured the full width at half maximum (FWHM) and the energy density of AzoPMA **3**. We found that the FWHM increased and the energy density decreased with increasing DCM (see Fig. 3b).

We used infrared (IR) imaging of a DSC pan containing ~11 mg of AzoPMA **3** on a hotplate (>100 °C) to visualize the enhanced exothermic isomerization in bulk samples of AzoPMA **3** dried from THF and DCM. IR images at ~1 s intervals for ~4 s are shown in Fig. 3c. Average IR intensity traces for each time-frame are shown in Fig. 3d. After ~4 s, the IR intensity of samples from DCM increased gradually. However, for samples dried from THF the IR intensity gradually increased after ~3 s, and then after ~4 s a large burst of IR intensity is observed (designated by an arrow in Fig. 3d). This burst of IR intensity is due to the heat released from the sample during *Z*-*E* isomerization. The differences in the energy density coupled with the differences in the shape of the DSC exotherm indicated the solvent used for processing AzoPMA polymers is critical to achieving high-energy densities.

### Solid-State Isomerization Kinetics

**Figure 4:**
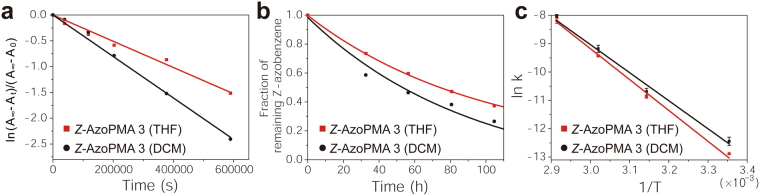
*Z*-*E* isomerization kinetics of solid AzoPMA **3** films dried from THF vs. DCM. (**a**) First-order graph of ln[*Z*-isomer] vs. time (s) for *Z*-AzoPMA **3** films dried from THF (red) or DCM (black) at 25 °C with corresponding linear fits. According to the first-order rate law, the slope of the fit is −*k* (s^−1^), the reaction rate coefficient. (**b**) Fraction of remaining *Z*-isomer population vs. time (**h**) for AzoPMA **3** dried from THF or DCM at 25 °C, with corresponding exponential fits. (**c**) Arrhenius plot of ln k vs. 1/T (K^−1^), for AzoPMA **3** dried from THF or DCM, with corresponding linear fits used to calculate *E*_a_. According to the Arrhenius equation the slope of the linear fit is *E*_*a*_/R.

We studied the solid-state *Z*-*E* isomerization kinetics using UV-Vis spectroscopy of AzoPMA **3** thin films fabricated from DCM or THF (see Fig. 4). At 25 °C, the rate coefficient of isomerization for films fabricated from THF was 2.55 (±0.07) × 10^−6^ s^−1^ (t_1/2_ = 75.5 h) and for films fabricated from DCM was 3.94 (±0.62) × 10^−6^ s^−1^ (t_1/2_ = 48.9 h). Moreover, the activation energy (*E*_*a*_) for films fabricated from THF was 91 ± 1.6 kJ/mol and for films fabricated from DCM was 82 ± 3 kJ/mol (see Fig. 4c). These results indicate that there are structural differences between the two films which lead to different rates of isomerization (discharging) in the solid-state, and thus achieving different physical properties from the same syndiotactic AzoPMA.

### Wide-angle X-ray Scattering

**Figure 5:**
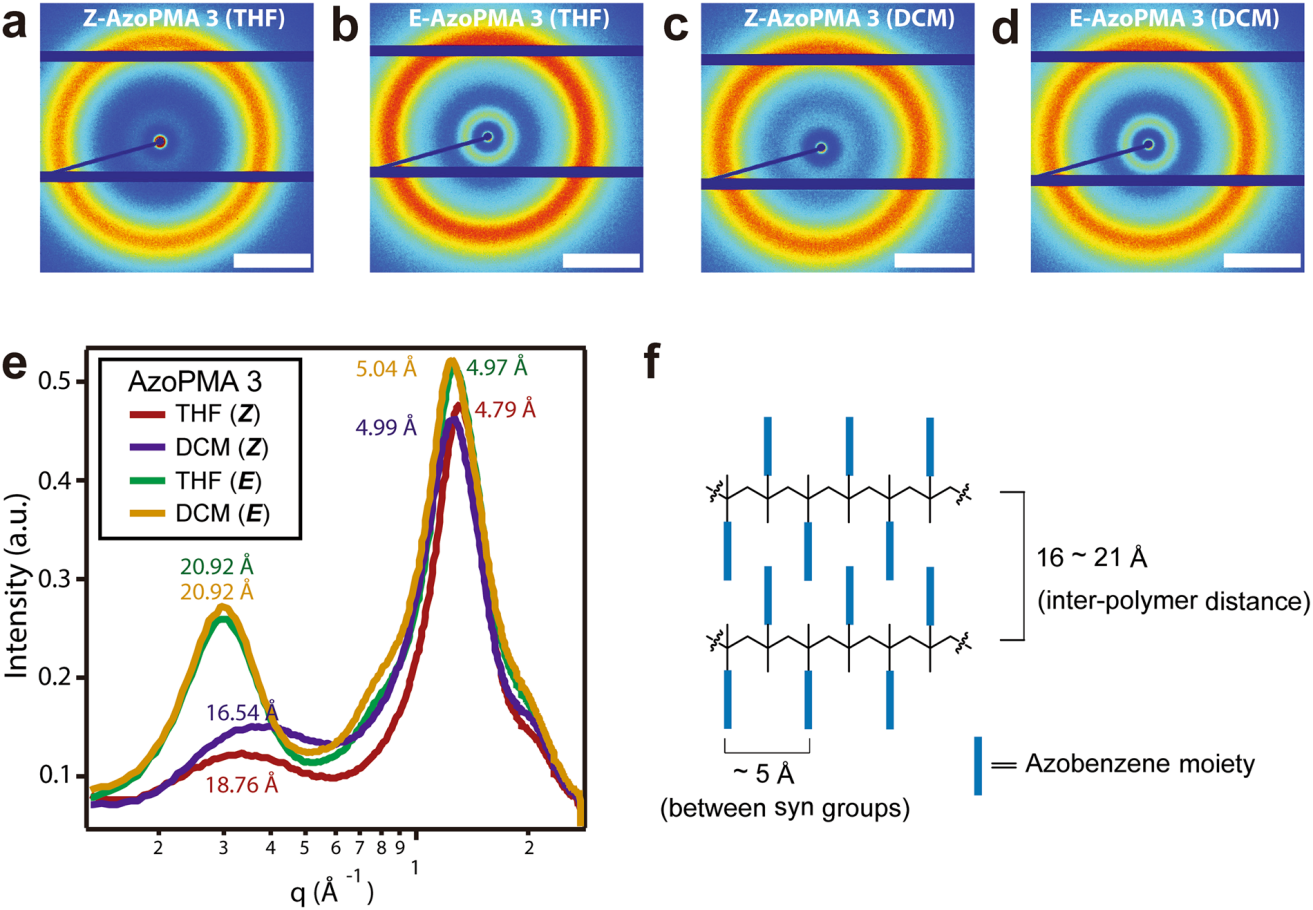
WAXS of solid AzoPMA **3** films dried from DCM vs. THF. 2-D WAXS patterns for (**a**) *Z*-AzoPMA **3** dried from THF, (**b**) *E*-AzoPMA **3** dried from THF, (**c**) *Z*-AzoPMA **3** dried from DCM, and (d) *E*-AzoPMA **3** dried from DCM; the scale bar is *q* = 1 Å^−1^. (**e**) Corresponding semi-log 1-D patterns of (**a**–**d**). Peaks at low *q* are attributed to inter-polymer packing, and peaks at higher *q* are attributed to syn triad spacing. (**f**) Representation of the distance between syn azobenzene groups (~5 Å) and the polymer-polymer packing distance (16–21 Å) in AzoPMA **3** polymer.

We used wide-angle x-ray scattering (WAXS) to characterize the bulk pressed pellets obtained from samples dried from DCM and THF, before and after UV irradiation (see Fig. 5). All WAXS spectra showed two peaks, the first at low *q* that is attributed to inter-polymer packing, and the second at higher *q* that is attributed to azobenzene syn triad spacing along a polymer chain. WAXS spectra showed syn triad spacing, with peaks from *q* = 1.25 Å^−1^ to 1.31 Å^−1^, which corresponds to a *d*-spacing of 5.04 Å to 4.79 Å, consistent with expectations of the distance between syn groups in a syndiotactic triad. This high *q* peak also has a shoulder at higher *q* which is attributed to the spacing between intercalated azobenzene units. In as-prepared samples with no UV treatment (*E*-AzoPMA), both samples showed similar *d*-spacing of 5.04 Å for DCM and 4.97 Å for THF. Upon UV irradiation, the *d*-spacing changes to 4.99 Å for DCM and 4.79 Å for THF. The shoulder attributed to intercalated azobenzene units is most prominent in *Z*-AzoPMA samples, and is most intense for samples dried from DCM. This result indicates a more closely assembled inter-chain packing in samples dried from DCM. The peaks at lower *q* are attributed to the distances between backbone of polymer chains. For samples dried from either solvent, prior to UV irradiation, the inter-polymer distance has an average *q* = 0.30 Å^−1^, which corresponds to a *d*-spacing of 20.92 Å. Upon UV irradiation, for samples from THF, the peak attributed to inter-polymer packing changes from *q* = 0.30 Å^−1^ to *q* = 0.33 Å^−1^ (*d* = 18.76 Å). For samples dried from DCM, the peak moved to *q* = 0.38 Å^−1^ (*d* = 16.54 Å), indicating a more compact packing of the polymer chains in this system.

### Kelvin Probe Force Microscopy

**Figure 6:**
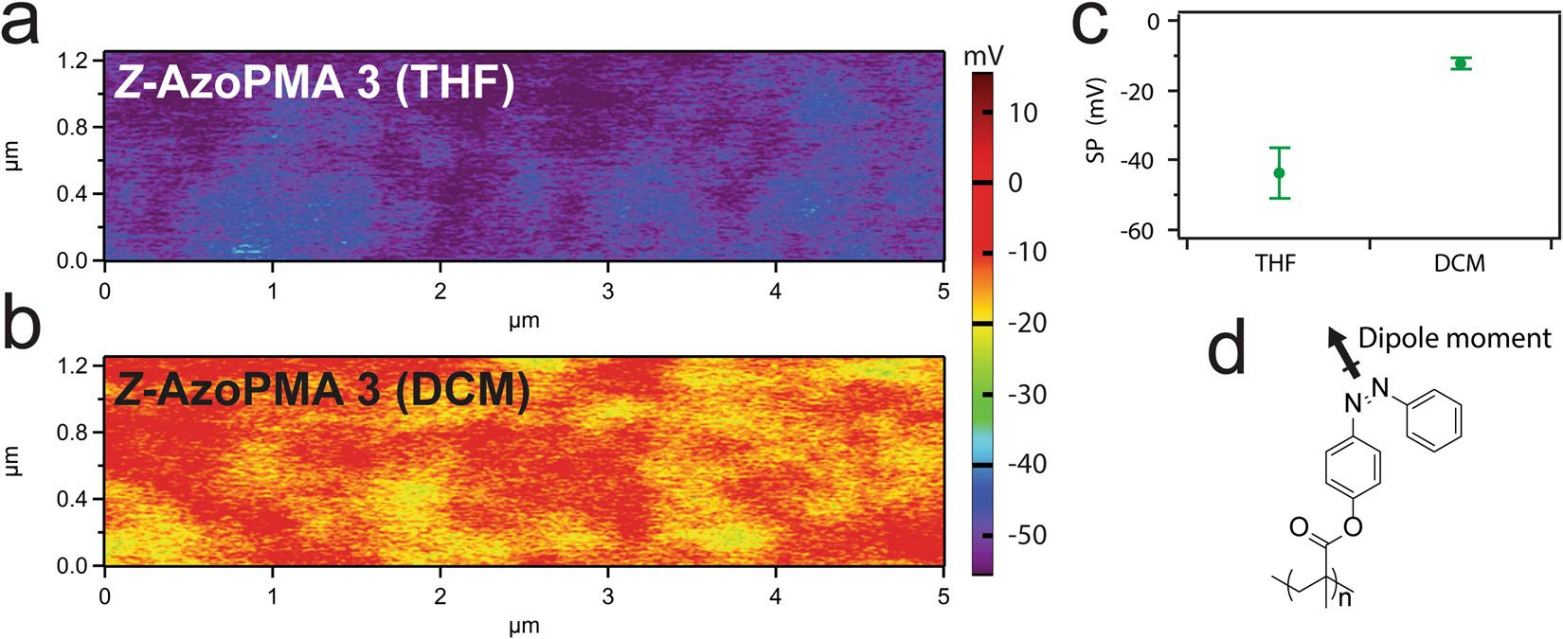
KPFM of solid AzoPMA **3** films from DCM and THF. KPFM surface potential maps of *Z*-AzoPMA **3** thin films on ITO glass spin-cast from (**a**) THF or (**b**) DCM. (**c**) Plot of thin film surface potential (SP, mV) vs. deposition solvent of *Z*-AzoPMA **3**. The data points represent the averages and standard deviations of SPs from three separate scans. (**d**) Chemical structure of *Z*-AzoPMA indicating the direction of dipole moment (~3 D).

Since the *Z*-isomer of the azobenzene has dipole moment of ~3 D^34^, we used Kelvin Probe Force Microscopy (KPFM) to probe any orientational preferences of the dipoles in films dried from THF and DCM^35,36^. Fig. 6a,b show representative surface potential maps for AzoPMA **3** spin cast from THF and DCM respectively. Fig. 6c shows the average surface potential (over three scans) for the two samples. The surface potential of solid thin films of *Z*-rich AzoPMA from the THF solution showed more negative value than the films of *Z*-rich AzoPMA from the DCM solution. Since solid-state UV-Vis measurements reveal that the *E*- to *Z*-isomer ratio is approximately the same, the surface potential differences indicate that the physical structure of two films prepared from same AzoPMA is different. The more negative surface potential of thin films of *Z*-rich AzoPMA prepared from THF solution implies that a larger fraction of dipole moments is aligned perpendicular to the surface compared to the DCM samples. On the other hand, we can assume that dipole moments of azobenzene units in *Z*-rich AzoPMA **3** prepared from DCM are cancelled by opposing dipole moments, possibly due to a more disorderly compact polymer structure. This is consistent with WAXS measurements which indicate that films from DCM have a higher degree of close inter-chain packing. From WAXS and KPFM results, we speculate that the reduced energy density in films dried from DCM is due to either the reduced space for isomerization, the disordered alignment of azobenzene moieties due to the compact packing, or a combination of both.

### Physical Properties of AzoPMA in Solution

#### Solution-State Isomerization Kinetics

**Figure 7:**
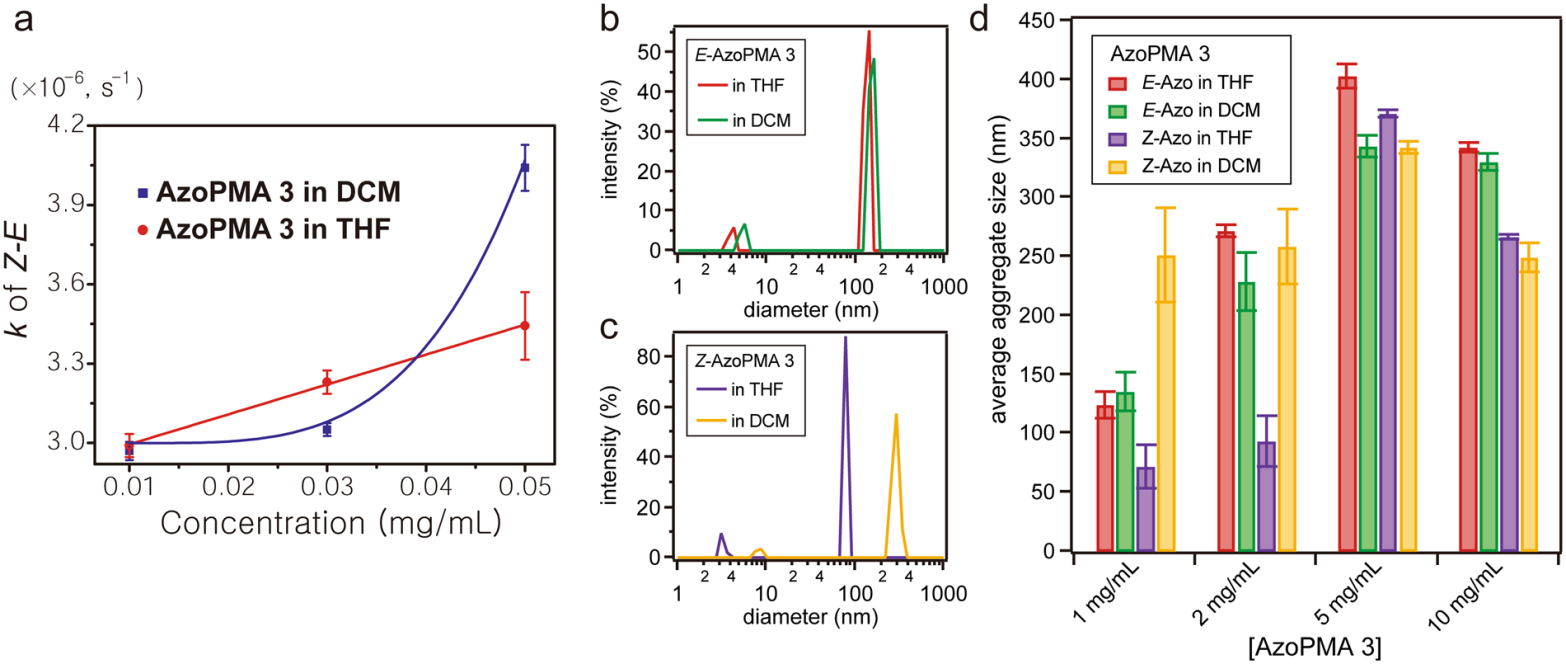
(**a**) Concentration dependent *Z*-*E* isomerization kinetics of AzoPMA **3** in THF and DCM. Graph of the *Z*-*E* isomerization rate coefficient (s^−1^) vs. AzoPMA **3** concentration (mg/mL) in THF (red marker) vs. DCM (blue marker). Data for THF is fit with a linear line, while DCM is fit with a power law equation. (**b**–**d**) DLS of AzoPMA **3** in THF and DCM. Representative semi-log plots of intensity (%) vs. diameter (nm) of (**b**) *E*-AzoPMA **3** (1 mg/mL) and (**c**) *Z*-AzoPMA **3** (1 mg/mL) in THF or DCM. (**d**) Plot of average aggregate size (from three measurements) vs. concentration of *E*- or *Z*-AzoPMA **3** in THF or DCM.

THF and DCM have a similar dipole moment and viscosity. We therefore expected that the rate coefficients for isomerization of AzoPMA **3** would be similar in these solvents. Contrary to our expectations, the rate coefficients of isomerization were quite different, particularly at higher polymer concentrations (Supplementary Fig. S26). The rate coefficients of isomerization, for AzoPMA **3** in THF, showed a linear increase with concentration. The rate coefficients of isomerization, for AzoPMA **3** in DCM, showed a marked non-linear increase with increasing polymer concentration, see Fig. 7a. This indicates that there is either a loss of stabilization of *Z*-isomer by the solvent or a cooperative effect that arises from increased concentration, or both^20^. As mentioned before, the polymer-solvent interaction parameter (*χ*) is −0.15 to 0.09 for poly(methylmethacrylate) (PMMA)-DCM and *χ* = 0.44 to 0.46 for PMMA-THF. The values indicate that PMA backbone has a stronger interaction with DCM than with THF^33^. Moreover, it is well-known that in strongly interacting systems, χ can decrease with increasing polymer concentration^37–39^. Therefore, we posit that the DCM solvent preferentially interacts with the PMA backbone over the azobenzene pendant groups of AzoPMA **3**, leading to the aggregation of the pendent azo-benzene units.

#### Dynamic Light Scattering

To confirm this hypothesis above, we looked at the aggregation of *E*- or *Z*-AzoPMA **3** with different concentrations using dynamic light scattering (DLS). For these studies, we used concentrations of 1 mg/mL, 2 mg/mL, 5 mg/mL, and 10 mg/mL in both solvents. Typical intensity percent vs. diameter DLS plots are shown in Fig. 7b,c for 1 mg/mL *E*- and *Z*-AzoPMA **3** respectively. All DLS curves show a first peak at <10 nm, which we attribute to individual polymer chains, followed by a second peak at ~50 nm–400 nm which we attribute to polymer aggregation. We note that the larger polymer aggregate peak is generally <1% of the volume percent, but is still a useful indicator of aggregation in these systems. The average aggregate size, over three measurements, for all concentrations, is plotted in Fig. 7d. When the polymer is in the *E* state, the polymer aggregate sizes are comparable at all measured concentrations. Conversely, in the *Z* state, 1 mg/mL polymer in THF displayed a relatively low aggregate size of 71.29 nm ± 18 nm, while the polymer in DCM had an average peak at 250.9 nm ± 40 nm. As the concentration of the polymer was increased to 2 mg/mL, the *Z* state aggregate peak in THF was still small compared to in DCM, increasing to 92.91 ± 22 nm and 258.5 ± 32 nm respectively. Upon further increase in the concentration, the aggregate size of the *Z*-isomer in THF and DCM were comparable to that in the *E*-isomer. These results support our hypothesis, based on polymer-solvent interaction parameter (*χ*), that DCM interacts more strongly with the PMA backbone, and thus does not solvate the *Z*-azobenzene moiety as effectively as THF. Furthermore, this reduced solvation and aggregation in relatively low concentrations of DCM, can lead to the more tightly packed inter-polymer spacing as we observed in WAXS, as well as less oriented alignment of dipoles observed in KPFM. Thus, we can conclude that a solvent that can solvate the *Z* state better, and does not have a very strong interaction with polymer backbone leads to more efficient and oriented packing of azobenzene units, giving a higher energy density.

### Isomerization Energy Calculation of AzoPMAs

We next turned to computational methods to gain further insights into the role of solvent and polymer morphology on the energy density.

#### Azobenzene Single Molecular System

**Figure 8:**
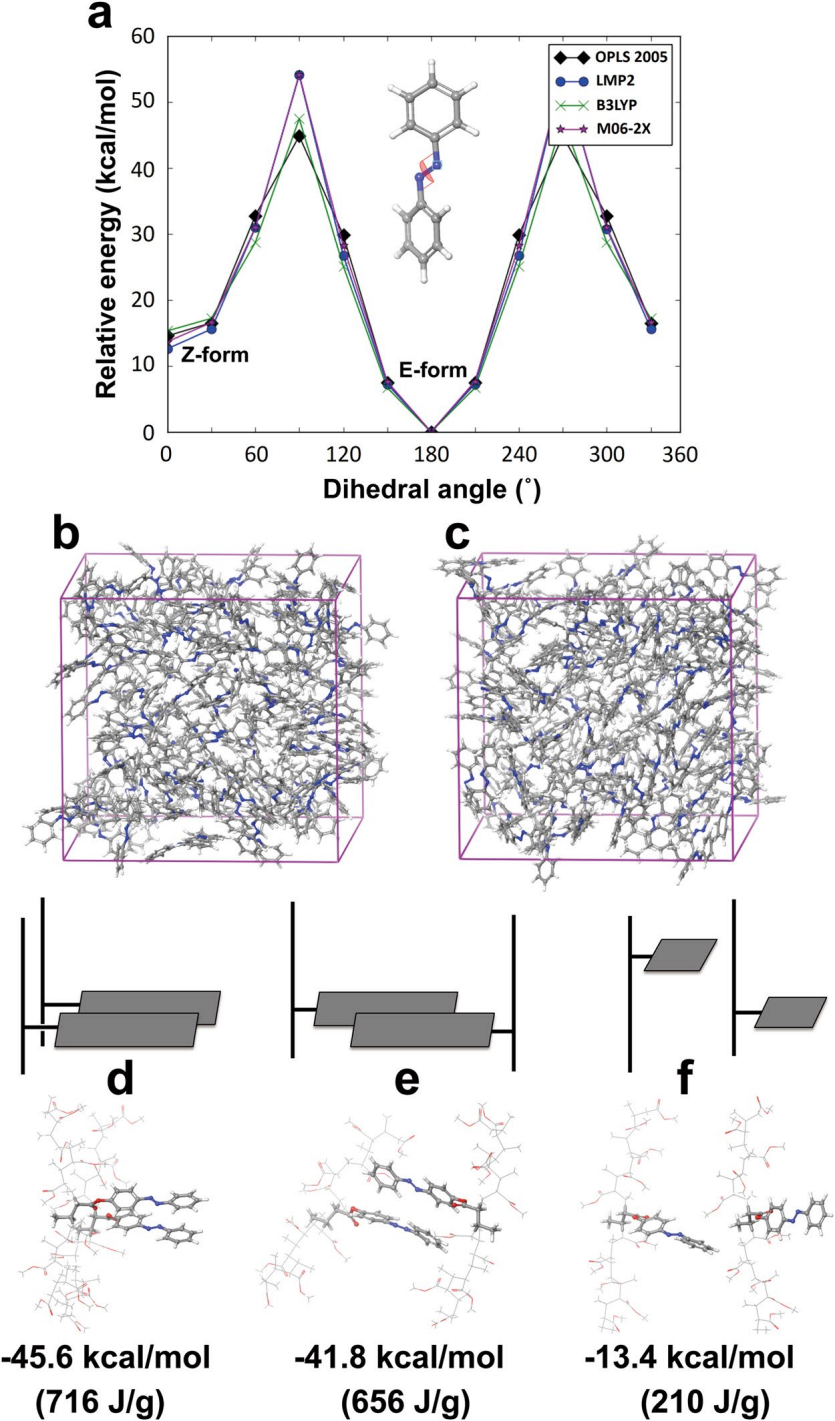
(**a**) Potential energy surface with respect to N-N dihedral angle of azobenzene computed from OPLS2005 force field in comparison to three different quantum chemical calculations using localized MP2 (LMP2) method and hybrid density functional theory (DFT) with B3LYP and M06-2X functionals, respectively. Illustration of simulation box for (**b**) *E*-isomeric and (**c**) *Z*-isomeric azobenzene in condensed-matter phase containing 125 molecules equilibrated at standard condition. (**d**–**f**) Illustrations (top) and snapshots (middle) of di-chain AzoPMA models where (**d**) both backbone chains and side-chain groups are in close contact each other, (**e**) only side-chain groups are in close contact while backbone chains are apart from each other, and (**f**) both backbone chains and side-chain groups are apart from one another. Theoretical predictions of isomerization energy per azobenzene group for the morphological representations (bottom) indicate the intermolecular interactions between side-chain (azobenzene) groups play the key role in determining the energetics of the isomerization.

We first analyzed the gas phase energy of this system. For a proper investigation of the side-chain *E*-*Z* isomerization from atomic scale, OPLS2005 force field^40^ was reconfigured and updated accordingly to accurately describe the potential energy surface of N-N dihedral angle in azobenzene groups. Fig. 8a illustrates the potential energy surface versus the N-N dihedral angle of an azobenzene molecule calculated with the modified OPLS2005 force field^40^ along with quantum chemical references—predictions from density functional theory with B3LYP^41,42^ and M06-2X^43^ hybrid functions as well as localized MP2 (LMP2) formalism^44^. As the Fig. 8a indicates, OPLS2005 force field can accurately describe not only the energy differences between *E*- and *Z*-isomers, but also the barrier between them to the scale of first-principles quantum chemical predictions. The consensus theoretical prediction is also consistent with experimental observation where the *E*-*Z* isomerization energy is measured to be −14 kcal/mol (321 J/g) and the barrier between the isomers to be about −37 kcal/mol (849 J/g)^45,46^.

Next we examined the solid-state effect over the *E*-*Z* isomerization by comparing the isomerization free energy of azobenzene at room temperature computed in gas phase to that of condensed-matter phase. To compute the isomerization free energy in condensed-matter phase, we built two 125-molecule boxes each with *E*- and *Z*-isomers (Fig. 8b,c) and equilibrated both at 1 atm and 300 K for 100 ps to compare the total energy. It was found that compared to the gas-phase isomerization free energy (−8.0 kcal/mol, 183 J/g), the solid-state isomerization free energy is nearly doubled (−14.8 kcal/mol, 340 J/g). The trend indicates there is a relatively stronger stabilization effect for *E*-isomers in the presence of intermolecular interactions when compared to the same effect for *Z*-isomers. It also provides a direct theoretical evidence of possibility in tuning such solid-state effect to control the energetics of isomerization in the materials containing azobenzene group.

#### AzoPMA Polymer System

Extending the theoretical analysis towards the AzoPMA system, three individual molecular models based on AzoPMA **3** were built for potential morphological representations in these materials (Fig. 8d–f). The models were set as di-chains in a simulation box of side-chain functionalized PMA backbone in different relative positions to represent simplified morphology after the solvent evaporation. Each of the three models corresponds to the case where backbone chains and/or side-chain groups in solid-state AzoPMA actively interact with one another. Each chain was built with eleven repeating units of methyl acrylate monomers, one of which is functionalized with an azobenzene group to represent the side-chain chemistry of AzoPMA. Conformation of the azobenzene group was set as either *E*- or *Z*-isomeric states before energy minimization of the entire di-chain model. *E*-*Z* isomerization energy was then sampled as the energy difference at 0 K originated from the two isomeric states within the model. As seen from the Fig. 8d–f, there is a drastic increase in the extent of isomerization energy when azobenzene groups are in close proximity (Fig. 8d,e). We also note that the extent of isomerization energies in these morphological representations (−41.8 kcal/mol and −45.6 kcal/mol) was nearly three-times larger than what was observed from an isolated azobenzene molecule (−14 kcal/mol). This can be explained that the molecular stabilization effect of the *E*-isomers is amplified by the polymer matrix when the side-chain groups are aligned to sit nearby at the *E*-isomeric conformation. On the other hand, the same effect does not hold for *Z*-isomers owing to the positional restraints over the azobenzene group by the backbone attachment, resulting in separation between the side-chain groups in *Z*-isomeric states. Also, relative positions of the backbone chains seem to have little effect over the energetics of *E*-*Z* isomerization. Observations from the molecular simulation and analysis of AzoPMA suggest the following: (1) Intermolecular interactions between the side-chain groups play important role in enhancing the energy density of AzoPMA, (2) π-stacking between the conjugated groups is likely the primary source of enhanced stabilization of the *E*-isomeric states, and (3) Relative positions of azobenzene groups through solvent dispersion could be the key in linking the solvent effect to the control of energy capacity.

### Glass Transition Temperature Difference

Glass transition temperature (*T*_*g*_) is related to free volume and polymer segmental motion^47^, and the calculation energy analysis reveals that the major reason for the high exotherm is the stabilization of the *E*-isomer state. Solvent processing with THF provides less polymer aggregation in a solution state, resulting in cooperative isomerization with high-energy density solids. Therefore, we assume that there is *T*_*g*_ difference between AzoPMA fabricated from DCM and THF in the second heating of DSC (after discharging). To test this, DSC measurement are set the same way as the previous one we set before. In the first heating curve, it is almost impossible to find *T*_*g*_ because heat release from *Z*-*E* isomerization starts before *T*_*g*_ (*T*_*g*_ = 62 °C) of pristine AzoPMA **3**. However, there is a *T*_*g*_ difference (~130 °C for DCM sample, ~90 °C for THF sample) between AzoPMAs fabricated from DCM vs. THF in the second heating of DSC (Supplementary Fig. S29). This indicates that the environment surrounding polymer strand is different. This is also consistent with the data from WAXS. Thus, we conclude that high free volume not only accelerates *Z*-*E* isomerization but also provides sufficient space for the *E*-isomer to have a strong stabilization through π-π stacking.

These studies indicate that *Z*-AzoPMA **3** films from THF pack in such a way, after isomerization, that the *E*-isomer can engage in π-π stacking. The stabilization can lead to a cooperative isomerization, which has been reported in polymers containing pendant azobenzene units^20^. In films obtained from DCM, there is insufficient space for the *E*-isomer to engage in π-π stacking. Our studies indicate that the origin of this packing difference is related to the nature of interaction between the solvent and the polymer backbone.

## Conclusion

We have demonstrated the fabrication of high-energy density azobenzene-based syndiotactic polymers. We have also demonstrated the critical role of polymer-solvent interactions on the fabrication of structures having a high-energy density. Different solvent processing can change physical polymer properties to tune energy density, heat-release shape, and activation energy of *Z*-*E* isomerization of AzoPMA materials. We find that if the solvent preferentially interacts with the polymer backbone, then it does not solvate the dipole formed in the charged *Z*-isomer, leading to aggregation in solution. We have shown that this aggregation in solution leads to a more compact inter-polymer packing, a more disorderly alignment of dipoles, and insufficient volume between the polymer backbones for π-π stacking between the pendant *E*-isomers after isomerization. These factors lead to a solid active layer that lacks cooperative isomerization, and thus lead to lower energy densities. Therefore, we determined that the processing solvent should sufficiently solvate the dipole formed in the charged (*Z*) state, in order to reduce aggregation in solution and lead to optimal structures that allows for efficient π-π stacking for high-energy density. Moreover, the theoretical investigation and the isomerization energy trend observed with respect to the morphological variation provides first-principles-based grounds to explain why and potentially how the solvent dispersion of AzoPMA materials would affect the *E*-*Z* isomerization energy in condensed-matter phases.

## Methods

### General Characterization Methods

^1^H NMR spectra were recorded on a 400 MHz Bruker Avance or 500 MHz Bruker Ascend NMR spectrometer, and ^13^C NMR spectra were proton decoupled and recorded on a 500 MHz Bruker Ascend NMR spectrometer using the carbon signal of the deuterated solvent as the internal standard. ^19^F NMR spectra were recorded on a 500 MHz Bruker Ascend NMR spectrometer. Chemical shifts are reported in parts per million (ppm) using the following abbreviations for peak multiplicities: s, singlet; d, doublet; t, triplet; m, multiplet; br, broad peak. Gel permeation chromatography (GPC) analyses were performed on an Agilent 1260 tetrahydrofuran (THF) GPC with polystyrene as a standard and toluene as the flow rate marker and RI detection mode. UV-Vis absorption spectra were measured using a Shimadzu UV 3600PC spectrometer or Shimadzu UV-2401PC spectrometer, and stock solutions were prepared in DCM or THF. The UV irradiation source for testing energy density of AzoPMA polymers was a 450 W Hanovia mercury arc lamp (Cat. #: 7825-34, ACE glass Inc.) or Black-Ray^®^ UV Bench lamp (XX-15L, UVP Inc.). The UV irradiation source for testing thermal isomerization and kinetics studies was a TLC lamp (Model UVGL-25) at long wave (~365 nm) irradiation. Thermogravimetric analysis (TGA) was performed on a TA instruments Q50. Dynamic light scattering (DLS) was determined by Nano-ZS (Malvern Inc.) Zetasizer. Differential scanning calorimetry (DSC) was performed on a TA instruments Q200. Field emission scanning electron microscopy (Magellan 400 L XHR-SEM) was operated to examine the morphology of solid-state AzoPMA films dried from DCM or THF on silicon substrate.

### Polymer Synthesis and Characterization

Details of the experimental procedures and spectroscopic analyses of AzoPMA **1**, **2**, and **3** in this manuscript can be found in Supplementary Fig. S1–15.

### Charging condition for energy density check

Solution samples in THF or DCM were charged using visible filtered 450 W UV radiation source (Hanovia mercury arc lamp, Cat. #: 7825-34, ACE glass Inc.) at 25 °C for at least 1 h or Black-Ray^®^ UV Bench lamp (XX-15L, UVP Inc.) at 25 °C for at least 12 h.

### Differential Scanning Calorimetry

Differential scanning calorimetry (DSC) was performed on a TA instruments Q200 to check energy density of AzoPMA. The process of DSC measurement is as follow: equilibrate at 0 °C, isothermal at 0 °C for 5 min, heat to 140 °C at 5 °C/min, isothermal at 140 °C for 5 min, cool to 10 °C at 5 °C/min, isothermal at 10 °C for 5 min, and then heat to 150 °C at 5 °C/min. N_2_ atmospheric condition was maintained at 50 mL/min throughout all DLS measurements. The average energy density value for AzoPMA **1** dried from THF is calculated from two samples, the average density value for AzoPMA **2** dried from THF is calculated from thirteen samples, the average energy density for AzoPMA **3** dried from THF is calculated from four samples, and the average energy density for AzoPMA **3** dried from DCM is calculated from three samples. The energy density of AzoPMA polymers is calculated by integration of the area under the exotherm peak in the first heating region through the software of “TA Instruments *Universal Analysis 2000*”.

### IR Imaging

IR imaging was conducted with infrared camera (Bullard Inc.) to check heat release from the solid-state *Z*-AzoPMA **3**. AzoPMA **3** in DCM or THF was charged using a Black-Ray^®^ UV Bench Lamp (XX-15L, UVP Inc.) with visible filter at a distance of 10 cm for overnight and then transferred to an aluminum foil-wrapped vial. The solvent was evaporated *in vaccuo* to get a solid-state sample. The soild AzoPMA **3** was then further dried under vacuum for 5 h. The sample (~11 mg) was hermetically sealed in Al DSC pans for IR imaging. Discharging of *Z*-AzoPMA **3** dried from THF or DCM was done on a pre-heated hot plate while monitoring with an infrared camera.

### Wide-Angle X-Ray Scattering

Solid AzoPMA **3** polymer powders dried from THF or DCM were pressed into pellets for WAXS characterization. Pure polymer pellets were measured with Ganesha SAXS-Lab system using Cu K-α radiation (0.154 nm), at a sample-detector distance of ~101 mm.

### Kelvin Probe Force Microscopy

KPFM measurements were made in air using Asylum Research MFPD-SA instrument and Pt/Ir coated silicon probe, used as receive from AppNano (ANSCM-PT). Measurements were carried out in a two-pass manner; in the first pass the probe is mechanically driven to measure topography, in the second pass the probe is driven at its AC voltage resonant frequency at height of 30 nm above the surface to surface potential. Mechanical oscillations from potential differences between the sample and the probe are canceled by an applied DC bias via a feedback loop. Three scans were performed on each sample at different locations, with a scan size of 5 μm × 1.25 μm (512 pixel × 128 pixel) at 0.5 Hz. Histogram plots of counts vs. potential were made for each scan, and fit with a Gaussian distribution to obtain the average and standard deviation of the potential for a particular scan. The potentials reported in the text are the averages and standard deviations of the three independent scan averages.

### UV-Vis Kinetics for Thermal *Z*-*E* Isomerization of AzoPMA **3** Solution

A solution of AzoPMA **3** in THF or DCM was taken and the UV-vis spectra were recorded to check thermal *Z*-*E* isomerization of AzoPMA **3** solution. The AzoPMA **3** solution in a vial was capped and sealed and exposed to UV light (~365 nm) in dark. The UV-vis spectra of the UV irradiated samples were periodically recorded over a week in dark. The vial was then sealed again and wrapped in an aluminum foil and placed in dark.

### UV-Vis Kinetics for Thermal *Z*-*E* Isomerization of Solid AzoPMA **3** Films

AzoPMA **3** dissolved in THF or DCM was exposed to UV light (~365 nm) in dark. A microscope glass slide (3″ × 1″ × 1 mm, VWR Inc.) was coated by drop casting of the charged AzoPMA **3** solution. After drying at room temperature for 20 min in dark, the glass slide was put to vacuum oven at room temperature for 10 min for further dry. The UV-vis spectra of the UV irradiated samples were periodically recorded over a week in dark. Three different samples from THF or DCM were made for calculating the rate coefficient of *Z*-*E* isomerization. The glass slides were placed in dark during entire UV-Vis measurement.

### Dynamic Light Scattering

DLS experiments were performed on a Malvern Nano-ZS Zetasizer using a 1 cm path length quartz cuvette. A solution of *E*-AzoPMA **3** (pristine AzoPMA **3**) in dried THF or DCM (1, 2, 5, and 10 mg/mL) was prepared, firmly sealed to prevent solvent from any evaporation, and then heated to 50 °C for 10 min in the dark to convert any residual *Z*-isomer to *E*-isomer. The samples were then cooled to 25 °C. After 12 h, the samples were tested by DLS. A solution of *Z*-AzoPMA **3** in dried THF or DCM (1, 2, 5, and 10 mg/mL) was prepared with ~365 nm UV irradiation (source: Black-Ray^®^ UV Bench lamp) for overnight from the stock solutions of *E*-AzoPMA **3** (1, 2, 5, and 10 mg/mL). All the samples were tested by DLS within 1 h after UV irradiation. Three scans were made for each sample. Intensity vs. diameter plots were used to determine aggregate size, volume vs. diameter plots were used to approximate the percent of aggregate. The temperature for DLS measurement was maintained at 25 °C throughout the experiment.

### Computational Calculation Methods

The entire computational work presented here, including quantum chemical calculations and force-field-based analyses, was performed utilizing *Schrödinger Materials Science Suite* (Version 2.2). Quantum chemical simulations for potential energy surfaces of *E*-*Z* isomerization were run by the suite’s quantum chemistry package *Jaguar* (Version 9.1)^48^. Force-field-based static and dynamic simulations were carried out using the suite’s molecular mechanics and molecular dynamics packages: MacroModel (Schrödinger Release 2016-4: MacroModel) and Desmond Molecular Dynamics System (Schrödinger Release 2016-4: Desmond Molecular Dynamics System, Maestro-Desmond Interoperability Tools), respectively. OPLS2005 force field was used to describe the structure-energy relationship throughout the force-field-based simulations^40,49^.

### Data availability

All data generated or analyzed during this study are included in this published article (and its Supplementary Information files).

## Electronic supplementary material

Supplementary Information

IR Video of Heat release
