# Supplementary material for: High Energy Density in Azobenzene-based Materials for Photo-Thermal Batteries via Controlled Polymer Architecture and Polymer-Solvent Interactions
Source: Sci Rep. 2017 Dec 19;7:17773. doi: 10.1038/s41598-017-17906-w (PMC5736555; doi:10.1038/s41598-017-17906-w)
Supplement: Supplementary file 1 — Supplementary Information [file 41598_2017_17906_MOESM1_ESM.pdf]

Supplementary Information (SI)

High Energy Density in Azobenzene-based Materials for Photo-Thermal Batteries via Controlled Polymer Architecture and Polymer-Solvent Interactions

Seung Pyo Jeong[1], Lawrence A. Renna[1], Connor J. Boyle[1], Hyunwook S. Kwak[2], Edward Harder[3], Wolfgang Damm[3] and Dhandapani Venkataraman[1,*]

[1]Department of Chemistry, University of Massachusetts Amherst, 710 North Pleasant Street, Amherst, Massachusetts 01003-9303, USA.
[2]Schrödinger, Inc., Cambridge, MA, 02142, USA
[3]Schrödinger, Inc., New York, NY, 10036, USA
*dv@chem.umass.edu

Materials

All starting chemicals and reagents were obtained from commercial sources and were used without further purification unless otherwise noted.

4-bromo-4'-hydroxybiphenyl (99%), 2-(2-chloroethoxy)tetrahydro-2H-pyran (96%), cesium carbonate (Cs_2CO_3, 99%), DMSO (99.7%), anhydrous sodium sulfate (Na_2SO_4), sodium bicarbonate ($NaHCO_3$), phenylacetylene (98%) was distilled under reduced pressure, 4-(phenylazo)phenol (95%), triethylamine (99%), bis(triphenyl phosphine) palladium(II) dichloride ($PdCl_2(PPh_3)_2$, 98%), copper(I) iodide (CuI, 98%), 2-bromo-2-methylpropionyl bromide (98%), methacryloyl chloride (97%), 2,2′-azobis(2-methylpropionitrile) (AIBN) (98%), tris[2-(dimethylamino)ethyl]amine (Me_6TREN, 97%), copper wire (Exeter Analytical Inc.), methanol, dichloromethane (DCM), tetrahydrofuran (THF), N,N-dimethylformamide (DMF), iron(III) chloride ($FeCl_3$), nitromethane ($MeNO_2$, 99%), poly(methyl methacrylate) (PMMA, Mw: 81K, Cat#: 7678, Polysciences Inc.).

S1. Polymer synthesis and characterization

S1.1. Synthesis of AzoPMA 1.

Experimental Procedure

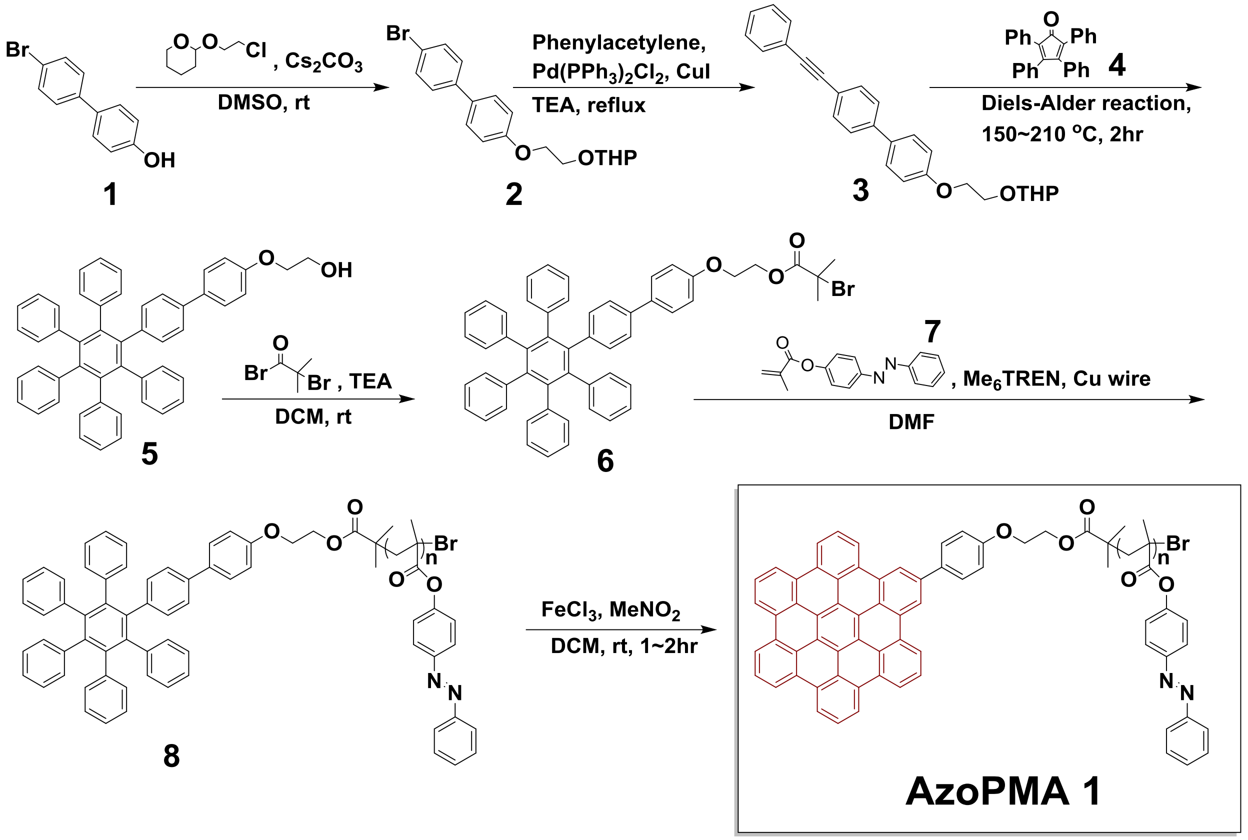

Supplementary Figure S1. Synthetic route of AzoPMA **1**.

Compound **1** was purchased and compound **2, 3, 4, 5,** and **6** were prepared according to literature procedure[1].

Synthesis of 4-(phenyldiazenyl)phenyl methacrylate, compound 7.

A solution of p-phenylazophenol (5.0 g, 25.2 mmol), triethylamine (3.73 mL, 26.7 mmol) in anhydrous DCM (80 mL) was slowly added to methacryloyl chloride (2.48 mL, 25.3 mmol) in a 100 mL Schlenk flask placed in an ice bath. The reaction mixture was stirred on the ice bath for 30 min and then for overnight at room temperature. The reaction mixture was then filtered and the filtrate was washed with distilled water and then brine before being dried over Na_2SO_4. The solvent was removed under reduced

pressure and the resulting mixture was purified by column chromatography on silica gel with Hexane/EA (20:1) to obtain **7** as a yellow colored powder (4.79 g, 71 %).

^{1}H NMR (400 MHz, $CDCl_3$): δ 7.99 (m, 2H), 7.92 (m, 2H), 7.50 (m, 3H), 7.31 (m, 2H), 6.39 (s, 1H), 5.80 (m, 1H), 2.09 (s, 3H).

^{13}C NMR (125 MHz, $CDCl_3$): δ 165.72, 153.12, 152.73, 150.37, 135.86, 131.26, 129.30, 127.92, 124.27, 123.05, 122.48, 18.61.

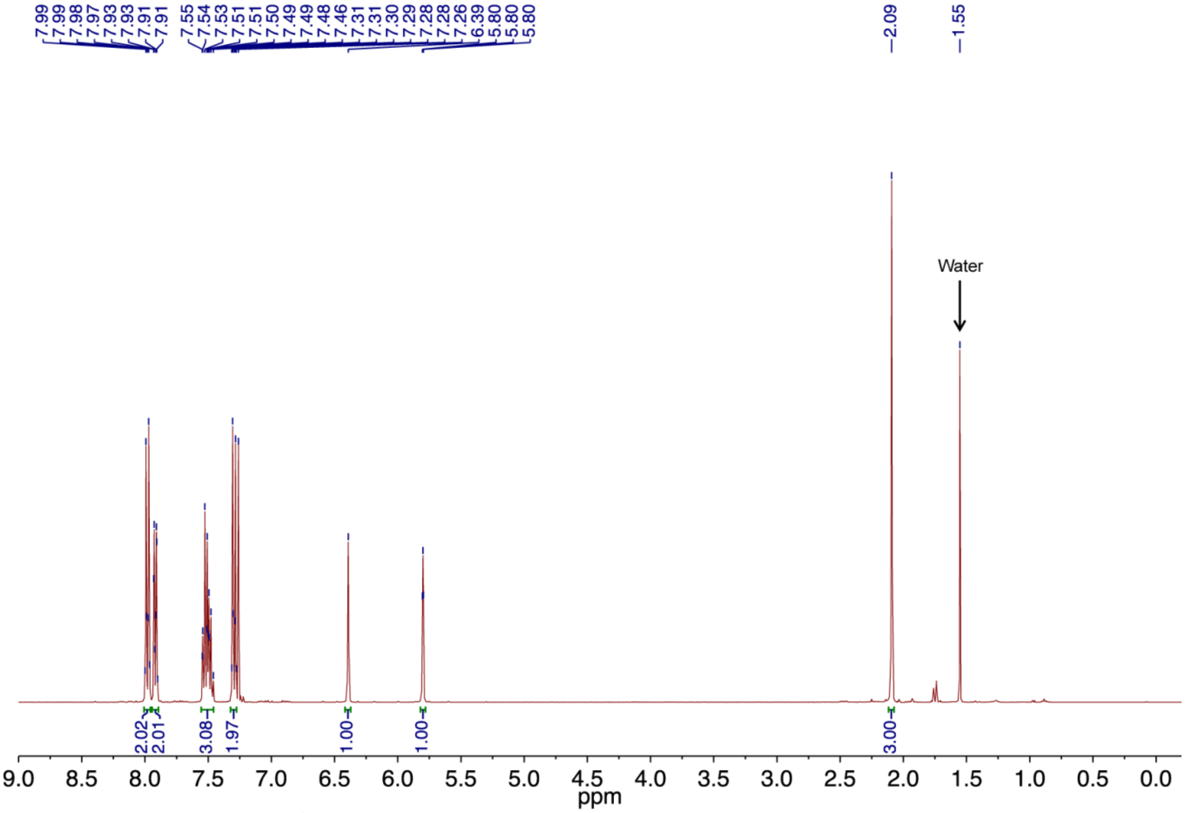

Supplementary Figure S2. ^{1}H NMR ($CDCl_3$) of 4-(phenyldiazenyl)phenyl methacrylate, compound 7.

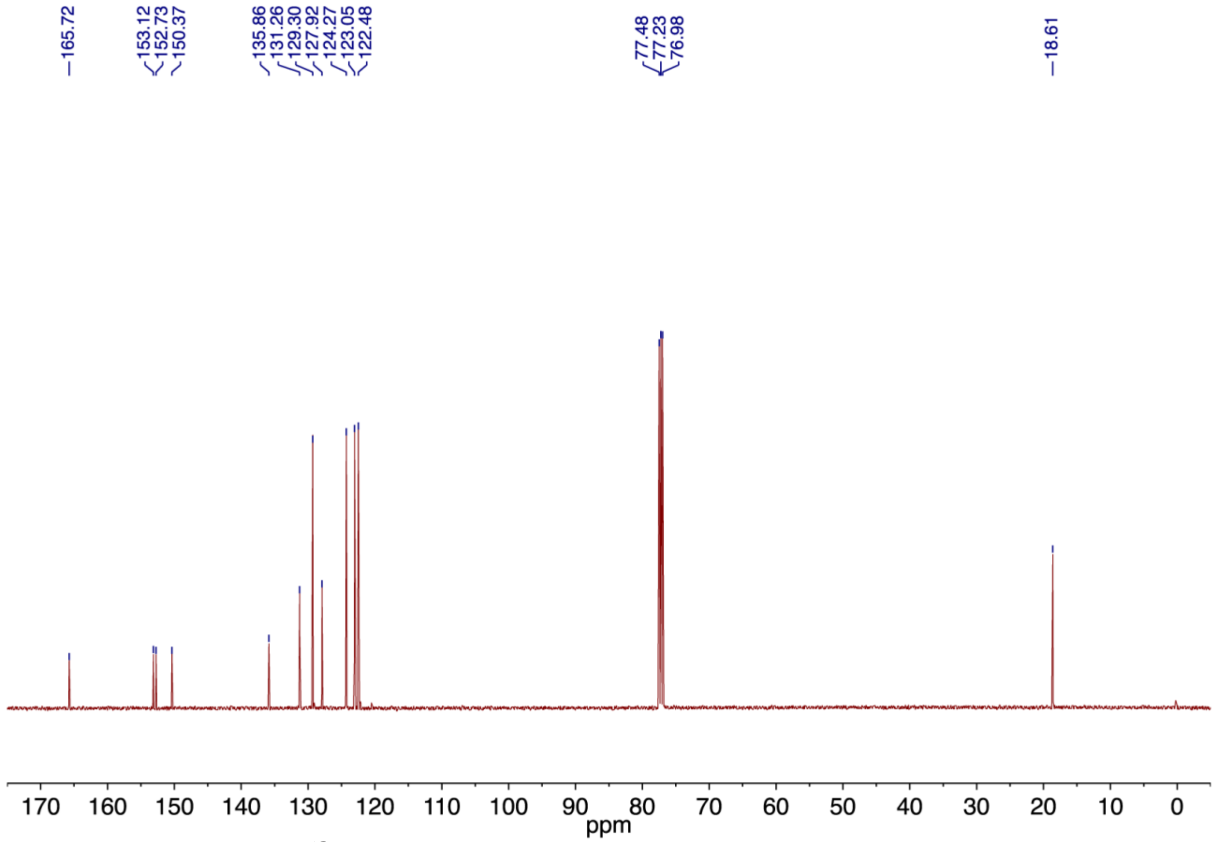

Supplementary Figure S3. ^{13}C NMR ($CDCl_3$) of 4-(phenyldiazenyl)phenyl methacrylate, compound 7.

Synthesis of AzoPMA end-functionalized with hexaphenylbenzene, compound 8.

A 100 mL Schlenk flask containing **6** (105 mg, 0.128 mmol), **7** (4.08 g, 15.3 mmol), and Cu wire (1.69 g) was degassed 3 times by the freeze-pump-thaw method. The contents of the flask were then stirred for 30 min until all chemicals were dissolved after adding DMF (degassed with nitrogen gas, 9.5 mL) by syringe. After dropping of Me_6TREN (0.5mL from 0.1M solution in DMSO, 0.005 mmol) by syringe, the contents of the flask were stirred at 50 °C for 90 min. The reaction mixture was poured into methanol to quench the reaction. The resulting precipitate was isolated by filtration, and washed with methanol several times, and dried under reduced pressure to obtain **8** as a brown/yellow powder (1.33 g, M_n: 16,798, M_w: 33,498, $Đ$: 1.99).

^1H NMR (400 MHz, $CDCl_3$): δ 7.69 – 7.99 (br), 7.31 – 7.51 (br), 7.14 - 7.29 (br), 6.83 (s, br), 4.41 (m), 4.10 (m), 2.17-2.75 (m, br), 1.86 – 2.05 (m, br), 1.16 – 1.81 (m, br).

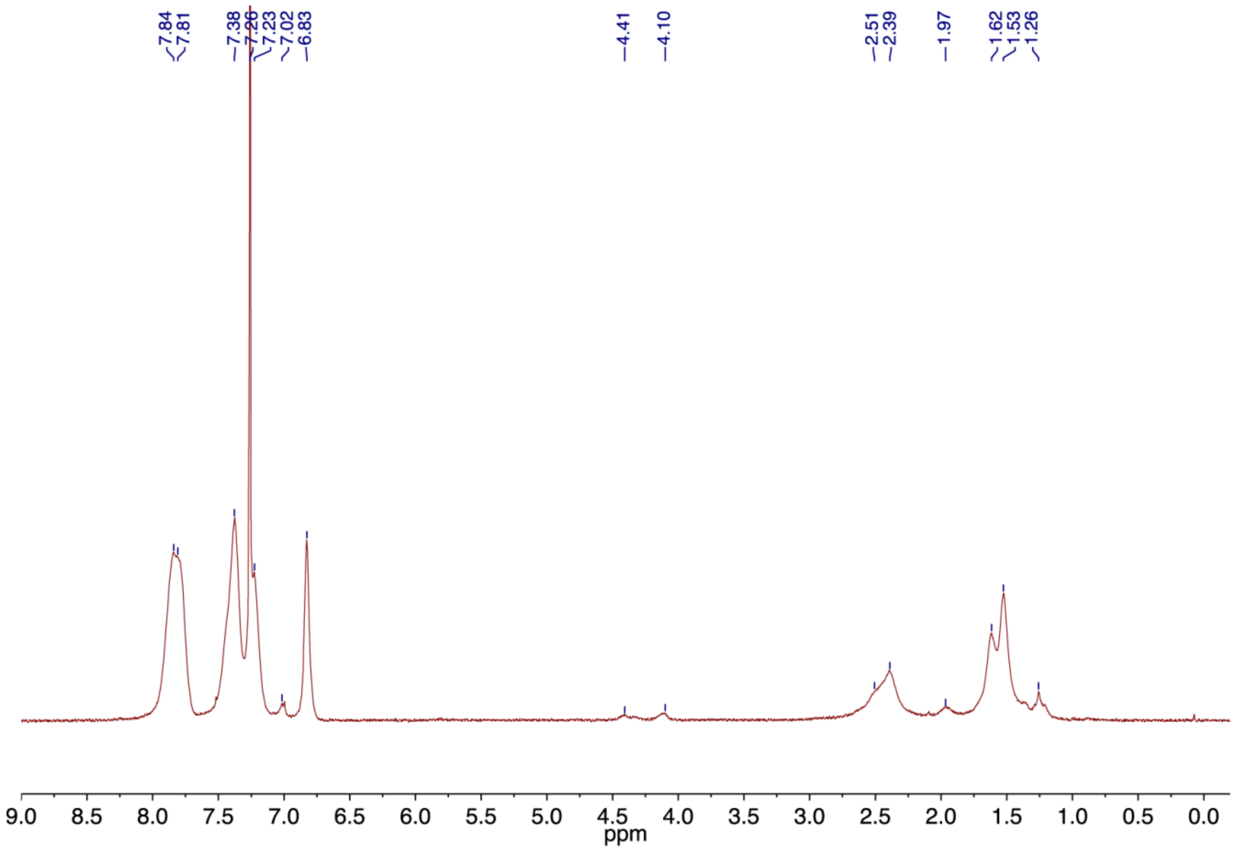

Supplementary Figure S4. ^{1}H NMR ($CDCl_3$) of AzoPMA end-functionalized with hexaphenylbenzene, compound 8.

Synthesis of AzoPMA 1.

*The detailed characterization of AzoPMA **1** will be published elsewhere.

The Scholl oxidation of compound **8** was carried out using a procedure slightly modified from the literature[2]. Anhydrous $FeCl_3$ (0.175 g, 1.08 mmol) was dissolved in nitromethane (1.57 mL) and this solution was slowly added to a solution of **8** (126 mg, ~0.0075 mmol) in anhydrous DCM (100 mL) in a 100 mL round bottom flask with argon bubbling through the solution using a glass capillary. An argon stream was bubbled through the reaction mixture for 2 h. The reaction mixture was poured into methanol (500 mL). The resulting yellow precipitate was isolated by filtration, and washed with methanol several times, and dried under reduced pressure to obtain AzoPMA **1** as a yellow brown powder (108 mg, M_n: 10,187, M_w: 24,325, $Đ$: 2.38).

^{1}H NMR (500 MHz, $CDCl_3$): δ 8.79 (br, very weak), 7.63 – 7.98 (br), 7.30 – 7.49 (br), 7.09 - 7.28 (br), 6.82 (m, weak), 2.17-2.69 (m, br), 1.85 – 2.04 (m, br), 1.15 – 1.83 (m, br).

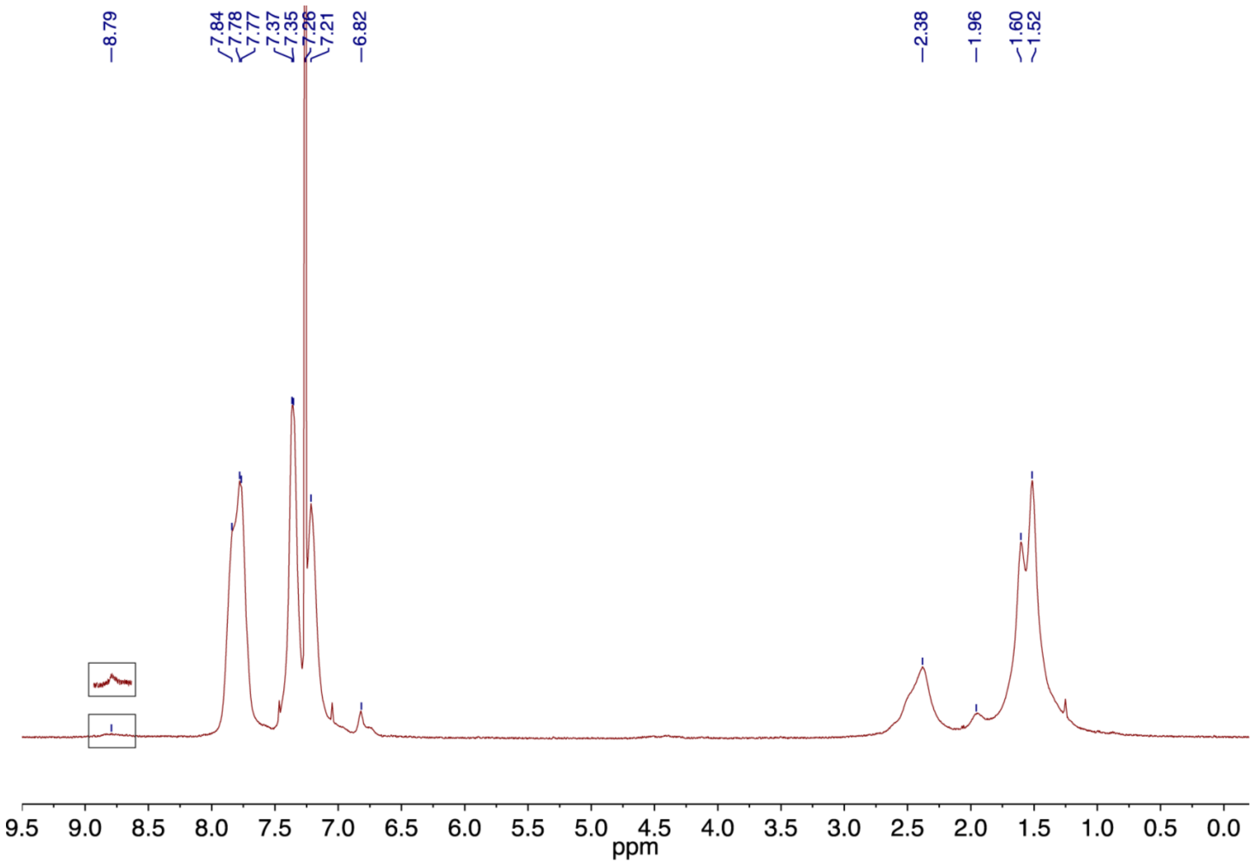

Supplementary Figure S5. ^{1}H NMR ($CDCl_3$) of AzoPMA **1**.

S1.2. Synthesis of AzoPMA 2.

Experimental Procedure

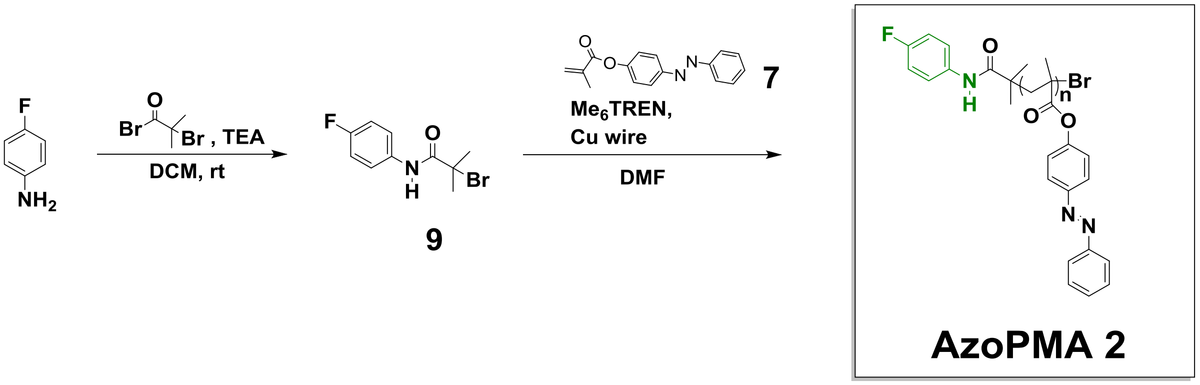

Supplementary Figure S6. Synthetic route of AzoPMA **2**.

Synthesis of 2-bromo-N-(4-fluorophenyl)-2-methylpropanamide, compound 9.

10 mL of DCM solution of 2-bromoisobutyryl bromide (1.12 mL, 9.0 mmol) was added slowly to a stirred solution of 4-fluoroaniline (1.0 g, 9.0 mmol) and triethylamine (1.28 mL, 9.23 mmol) in dry

DCM (30 mL) that was previously cooled (using an ice/water bath) and stirred over 30 min. After complete addition of 2-bromoisobutyryl bromide, the reaction was stirred at 0 °C for 30 min followed by stirring at room temperature for 10 h. The reaction mixture was filtered, then transferred into a separatory funnel and washed with water and then brine for 2 times before drying over Na_2SO_4 (~15 wt%) and removal of the solvent by rotary evaporation. The isolated solids, crude **9**, were purified by recrystallization in hexane to obtain **9** as colorless needle-like crystals (2.01 g, 85%). cf) Compound 9 is known compound.

^{1}H NMR (400 MHz, $CDCl_3$): δ 8.45 (s, br, 1H), 7.49 (m, 2H), 7.03 (m, 2H), 2.03 (s, 6H).

^{13}C NMR (100 MHz, $CDCl_3$): δ 170.19, 159.84 (d, J = 244.3 Hz), 133.52 (d, J = 2.9 Hz), 122.11 (d, J = 8.0 Hz), 115.86 (d, J = 22.5 Hz), 63.10, 32.67.

^{19}F NMR (471 MHz, $CDCl_3$): δ -117.14 (tt, J = 8.5, 4.7 Hz)

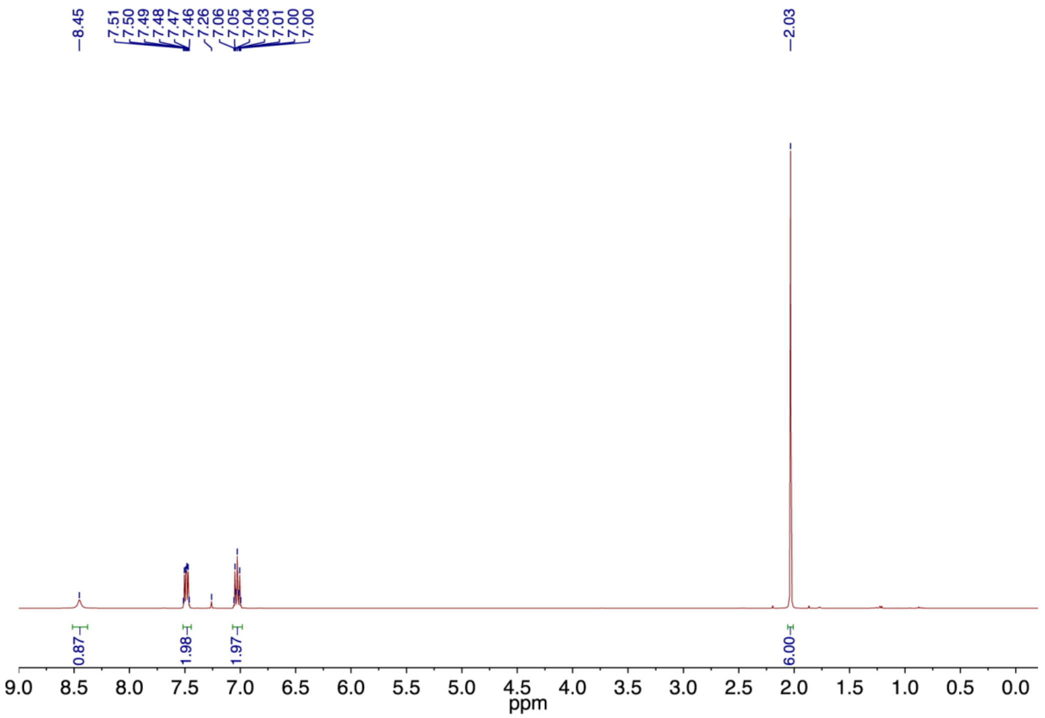

Supplementary Figure S7. ^{1}H NMR ($CDCl_3$) of 2-bromo-N-(4-fluorophenyl)-2-methylpropanamide, compound 9.

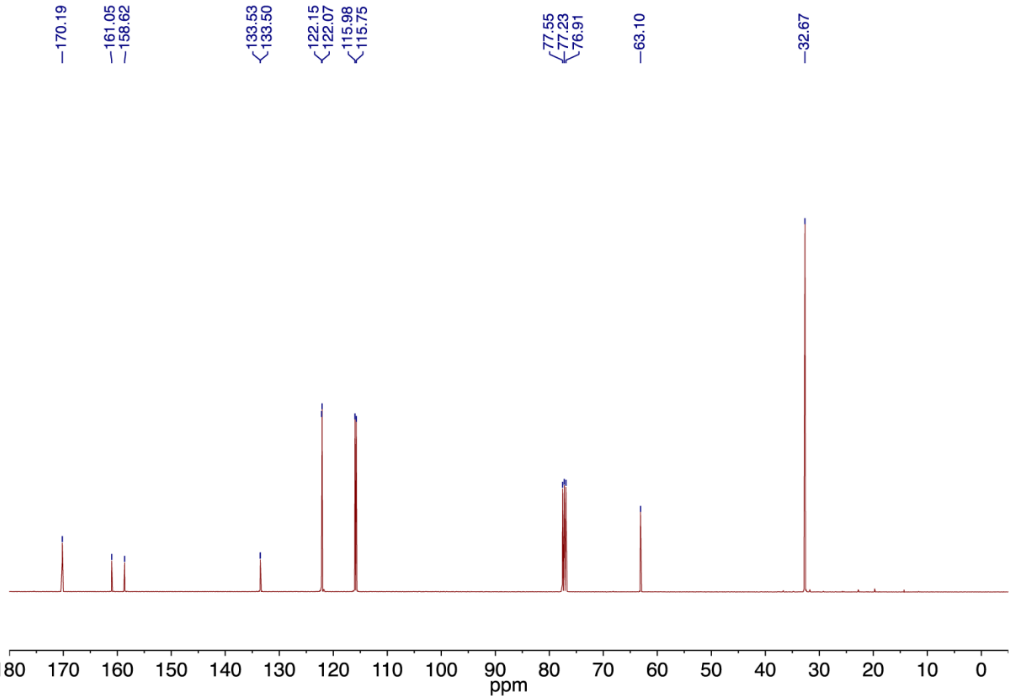

Supplementary Figure S8. ^{13}C NMR ($CDCl_3$) of 2-bromo-N-(4-fluorophenyl)-2-methylpropanamide, compound 9.

Synthesis of AzoPMA 2.

A 100 mL Schlenk flask containing **9** (17 mg, 0.065 mmol), **7** (1.2 g, 4.5 mmol), and Cu wire (0.85 g) was degassed 3 times using freeze-pump-thaw method. The contents of the flask were then stirred for 30 min until all chemicals were dissolved after adding DMF (degassed, 4 mL) by syringe. After dropping of Me_6TREN (0.1 mL from 0.1 M solution in DMSO, 0.01 mmol) by syringe, the contents of the flask were stirred for 4 h at 40 °C. The reaction mixture was poured into methanol (500 mL) to quench the reaction. The resulting precipitate was isolated by filtration, and washed with methanol several times, and dried under reduced pressure to obtain AzoPMA **2** as a yellow powder (0.42 g, M_n: 14,782, M_w: 25,895, *Đ*: 1.75).

^1H NMR (400 MHz, $CDCl_3$): δ 7.66 – 8.06 (br), 7.30 – 7.55 (br), 7.07 - 7.29 (br), 2.17-2.76 (m, br), 1.85 – 2.06 (m, br), 1.17 – 1.79 (m, br).

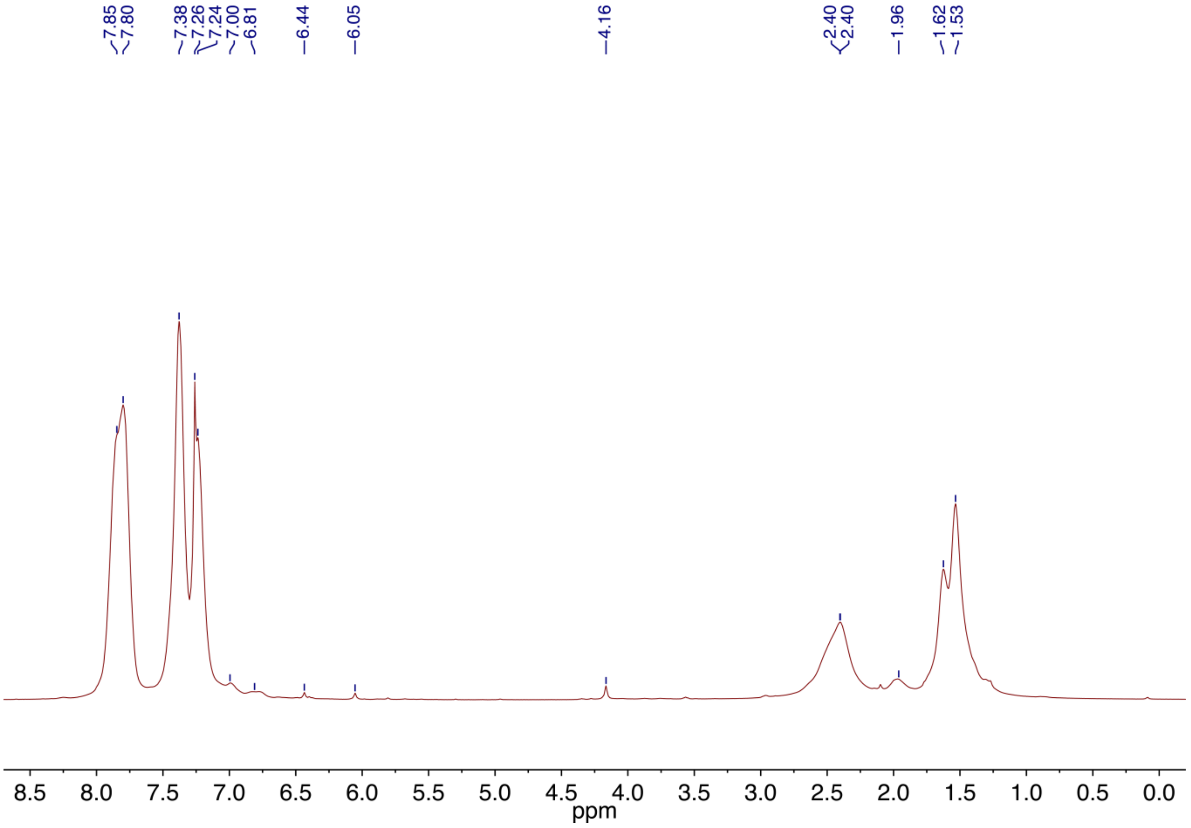

Supplementary Figure S9. ^{1}H NMR ($CDCl_3$) of AzoPMA **2**.

S1.3. Synthesis of AzoPMA 3.

Experimental Procedure

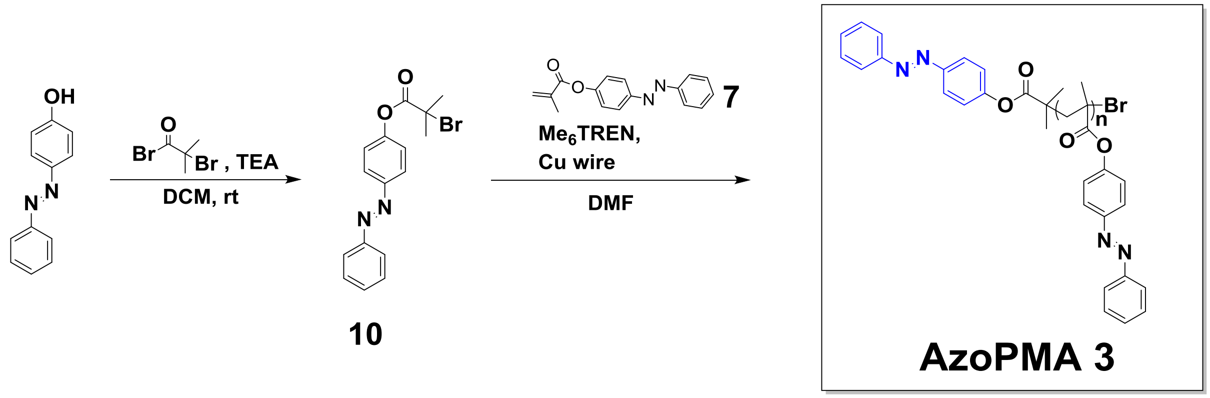

Supplementary Figure S10. Synthetic route of AzoPMA **3**.

# Synthesis of (E)-4-(phenyldiazenyl)phenyl 2-bromo-2-methylpropanoate, compound 10.

20 mL of DCM solution of 2-bromoisobutyryl bromide (1.25 mL, 10.17 mmol) was added slowly to a stirred solution of (E)-4-(phenyldiazenyl)phenol (1.68 g, 8.48 mmol) and triethylamine (1.42 mL, 10.17 mmol) in dry DCM (100 mL) that was previously cooled (using an ice/water bath) and stirred over 30 min. After complete addition of 2-bromoisobutyryl bromide, the reaction was stirred at 0 °C for 2 h and then stirred at room temperature for 24 h. The reaction mixture was washed with water 3 times before drying over Na_2SO_4 (~15 wt%) and removal of the solvent by rotary evaporation. The resulting mixture was purified by column chromatography on silica gel with hexane/ethyl acetate (30:1) to obtain **10** as a yellow colored powder (1.64 g, 56 %). Compound 10 was prepared according to literature procedure[3].

^1H NMR (500 MHz, $CDCl_3$): δ 7.99 (m, 2H), 7.92 (m, 2H), 7.52 (m, 3H), 7.30 (m, 2H), 2.10 (s, 6H).

^{13}C NMR (125 MHz, $CDCl_3$): δ 170.19, 152.82, 152.69, 150.65, 131.38, 129.33, 124.34, 123.10, 121.98, 55.42, 30.81.

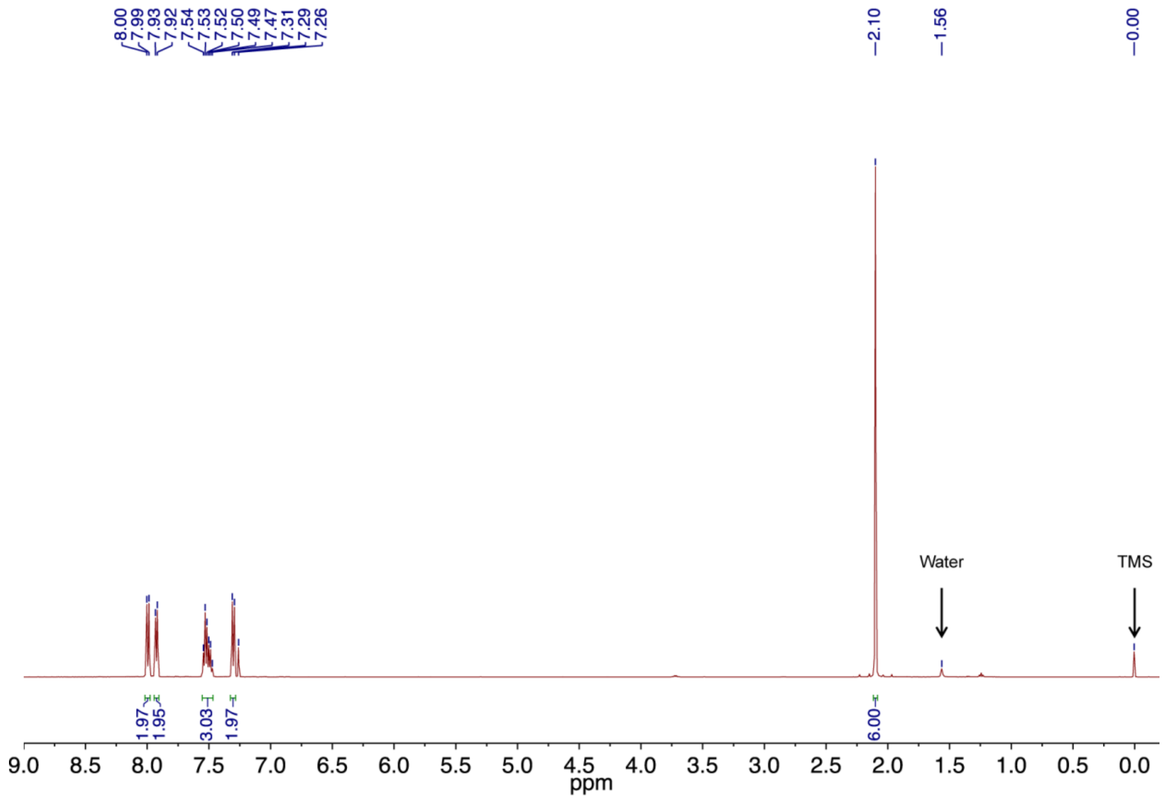

Supplementary Figure S11. ^1H NMR ($CDCl_3$) of (E)-4-(phenyldiazenyl)phenyl 2-bromo-2-methylpropanoate, compound **10**.

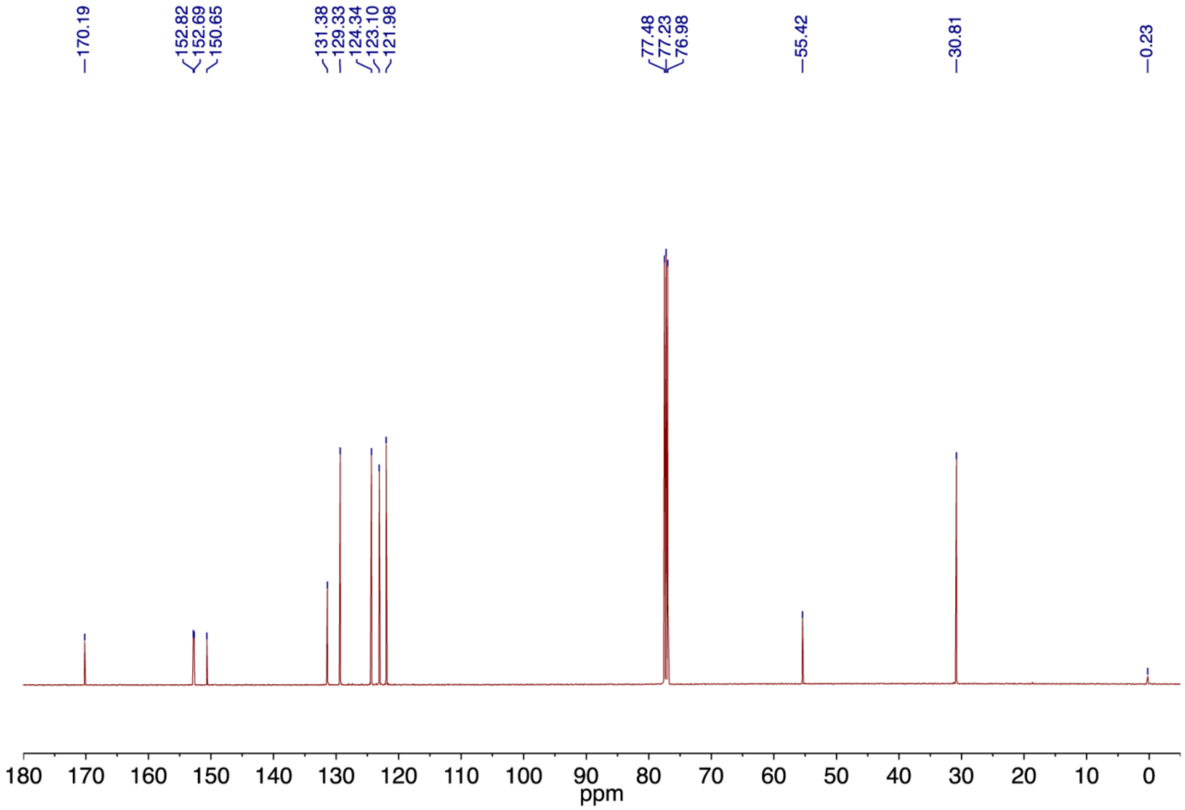

Supplementary Figure S12. ^{13}C NMR ($CDCl_3$) of (E)-4-(phenyldiazenyl)phenyl 2-bromo-2-methylpropanoate, compound 10.

Synthesis of AzoPMA 3.

A 100 mL Schlenk flask containing **10** (23.1 mg, 0.066 mmol), **7** (1.58 g, 5.93 mmol), and Cu wire (1.03 g) was degassed 3 times by the freeze-pump-thaw method. The contents of the flask was then stirred for 30 min until all chemicals were dissolved after adding 6 mL of degassed DMF by syringe. After dropping of Me_6TREN (0.1 mL from 0.1 M solution in DMSO, 0.01 mmol) by syringe, the contents of the flask were stirred for 6 h at 40 °C. The reaction mixture was poured into methanol (500 mL) to quench the reaction. The resulting precipitate was isolated by filtration, washed with methanol several times, and dried under reduced pressure to obtain AzoPMA **3** as a yellow powder (0.65 g, M_n: 14,811, M_w: 25,745, $Đ$: 1.73).

^1H NMR (500 MHz, $CDCl_3$): δ 7.64 – 8.07 (br), 7.30 – 7.56 (br), 7.06 - 7.29 (br), 2.19-2.76 (m, br), 1.82 – 2.06 (m, br), 1.19 – 1.79 (m, br).

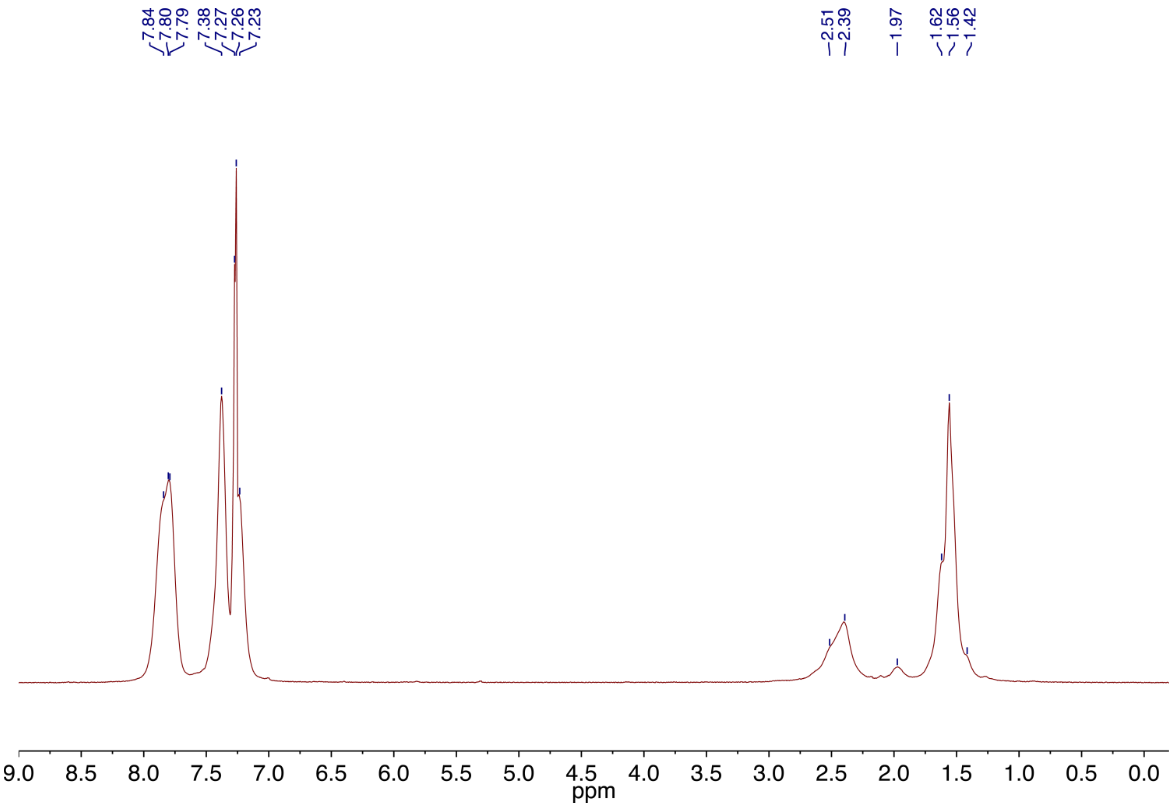

Supplementary Figure S13. ^{1}H NMR ($CDCl_3$) of AzoPMA **3**.

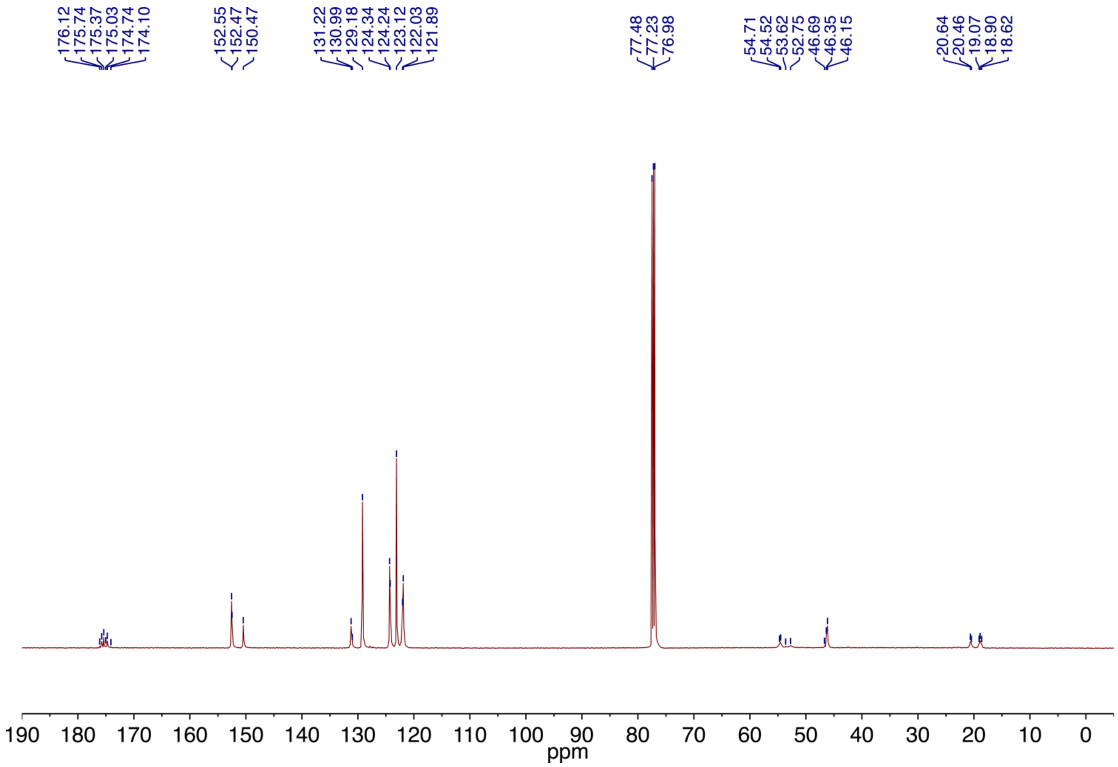

Supplementary Figure S14. ^{13}C NMR ($CDCl_3$) of AzoPMA **3**.

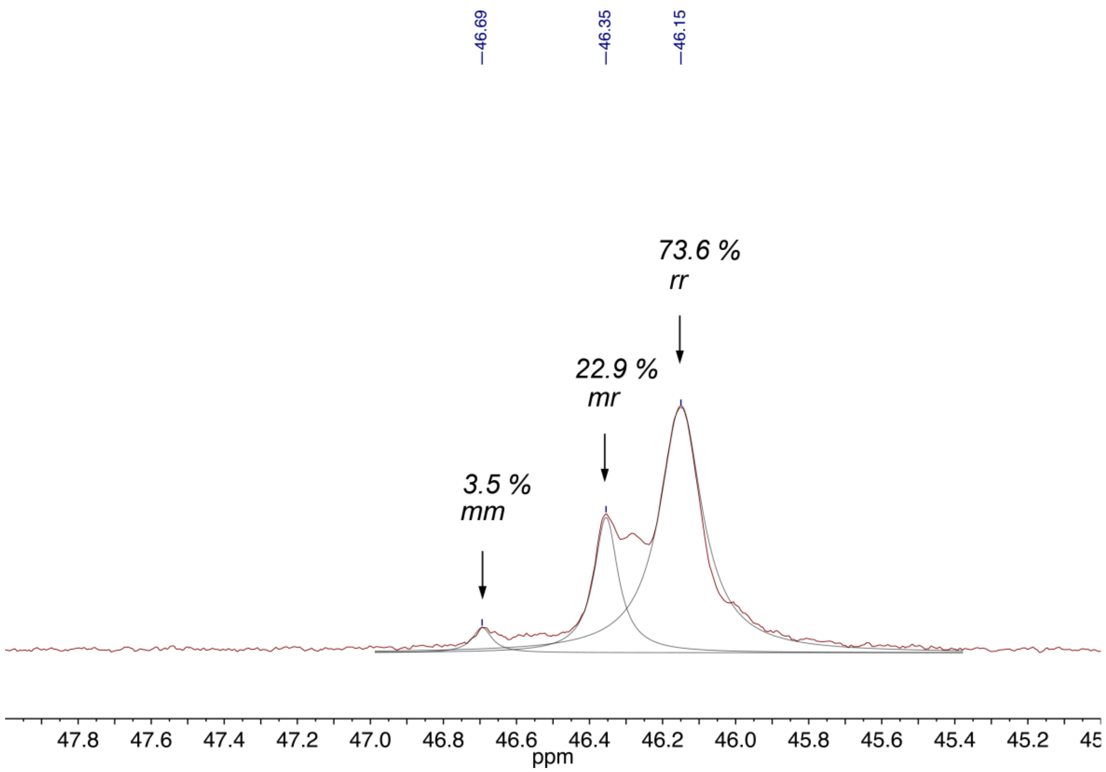

Supplementary Figure S15. Tacticity calculation of AzoPMA **3** from the ^{13}C NMR of AzoPMA **3**.

S1.4. Synthesis of AzoPMA 4.

Experimental Procedure

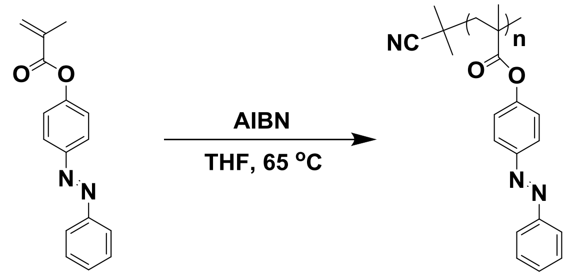

7 **AzoPMA 4**

Supplementary Figure S16. Synthetic route of AzoPMA **4**.

A 100 mL Schlenk flask containing **7** (1.02 g, 3.83 mmol) and AIBN (12 mg, 0.073 mmol) was degassed 3 times by the freeze-pump-thaw method. After adding dry THF (10 mL) by syringe, the contents of the flask were stirred for 6 h at 65 °C. The reaction mixture was poured into methanol (500mL) to quench the reaction. The resulting precipitate was isolated by filtration, washed with

methanol for several times, and dried under reduced pressure to obtain AzoPMA **4** as a yellow powder (0.46 g, M_n: 12,842, M_w: 21,446, *Đ*: 1.67).

^{1}H NMR (500 MHz, $CDCl_3$): δ 7.69 – 8.08 (br), 7.30 – 7.58 (br), 7.10 - 7.29 (br), 2.20-2.77 (m, br), 1.80 – 2.06 (m, br), 1.20 – 1.79 (m, br).

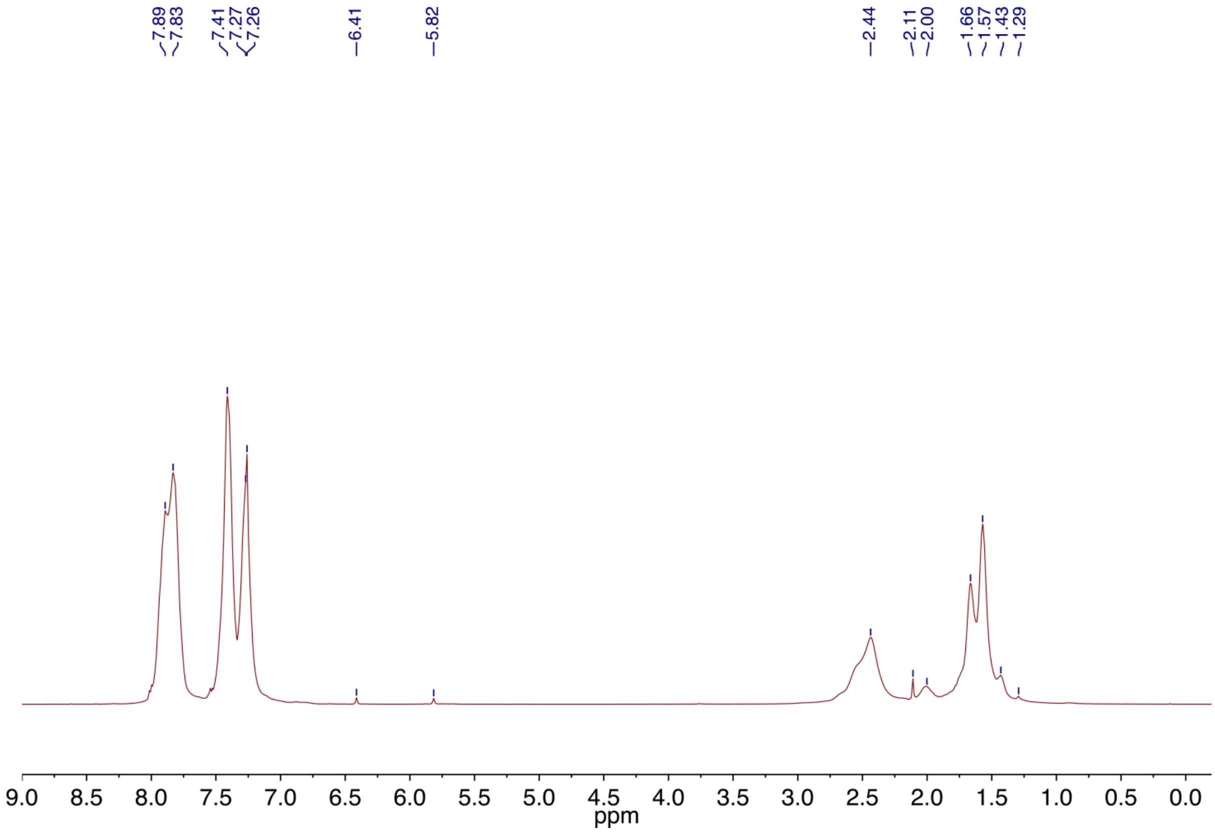

Supplementary Figure S17. ^{1}H NMR ($CDCl_3$) of AzoPMA **4**.

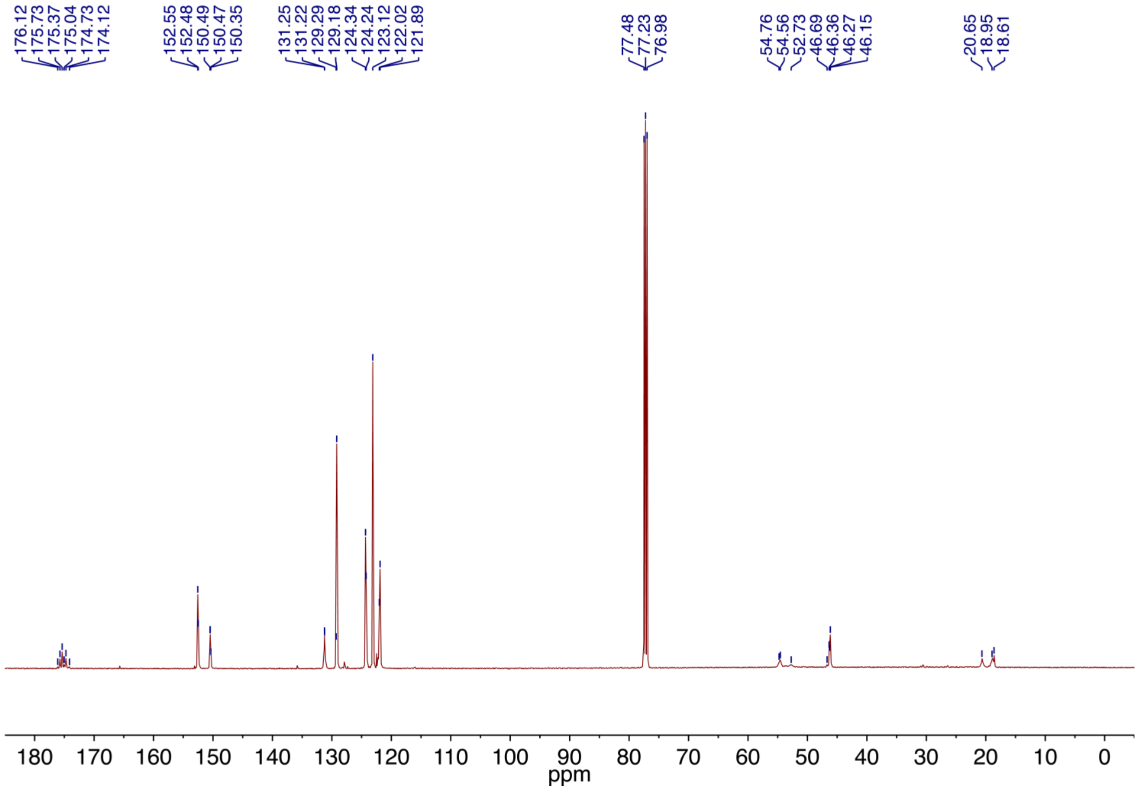

Supplementary Figure S18. ^{13}C NMR ($CDCl_3$) of AzoPMA **4**.

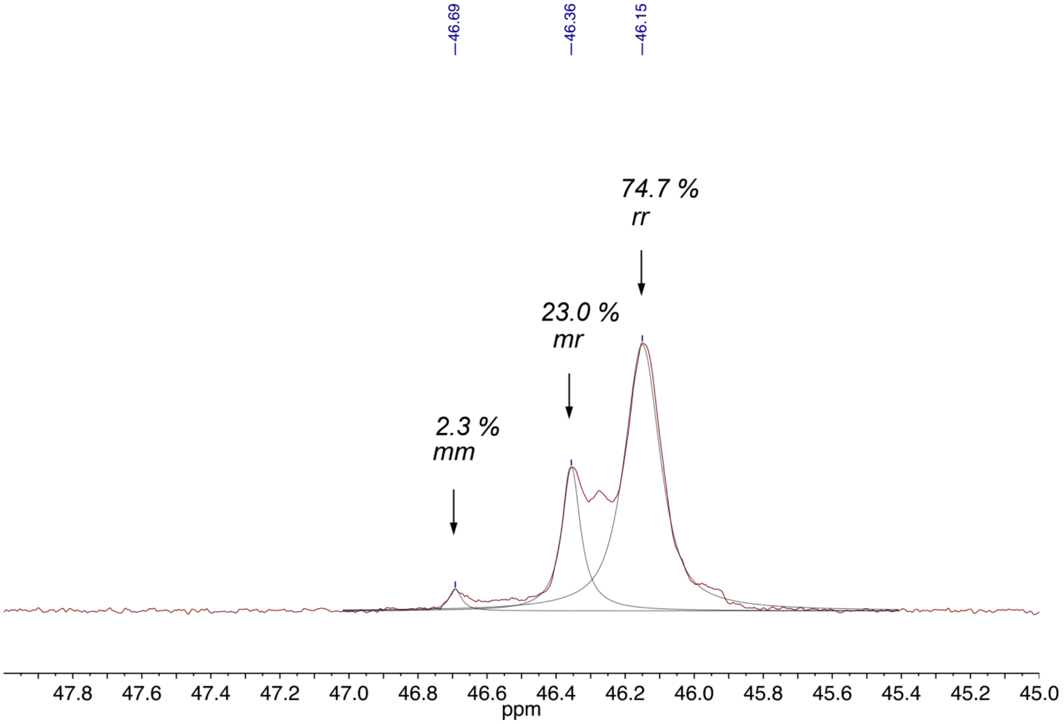

Supplementary Figure S19. Tacticity calculation of AzoPMA **4** from the ^{13}C NMR of AzoPMA **4**.

S2. Determination of thermal decomposition temperature of AzoPMA by TGA.

The thermal decomposition temperature of AzoPMA **1**, **2**, and **3** are determined by TGA. The sample chamber was maintained under N_2 gas atmosphere (60 mL/min). The sample was held at 25 °C, and then heated to 800 °C at a rate of 10 °C/min.

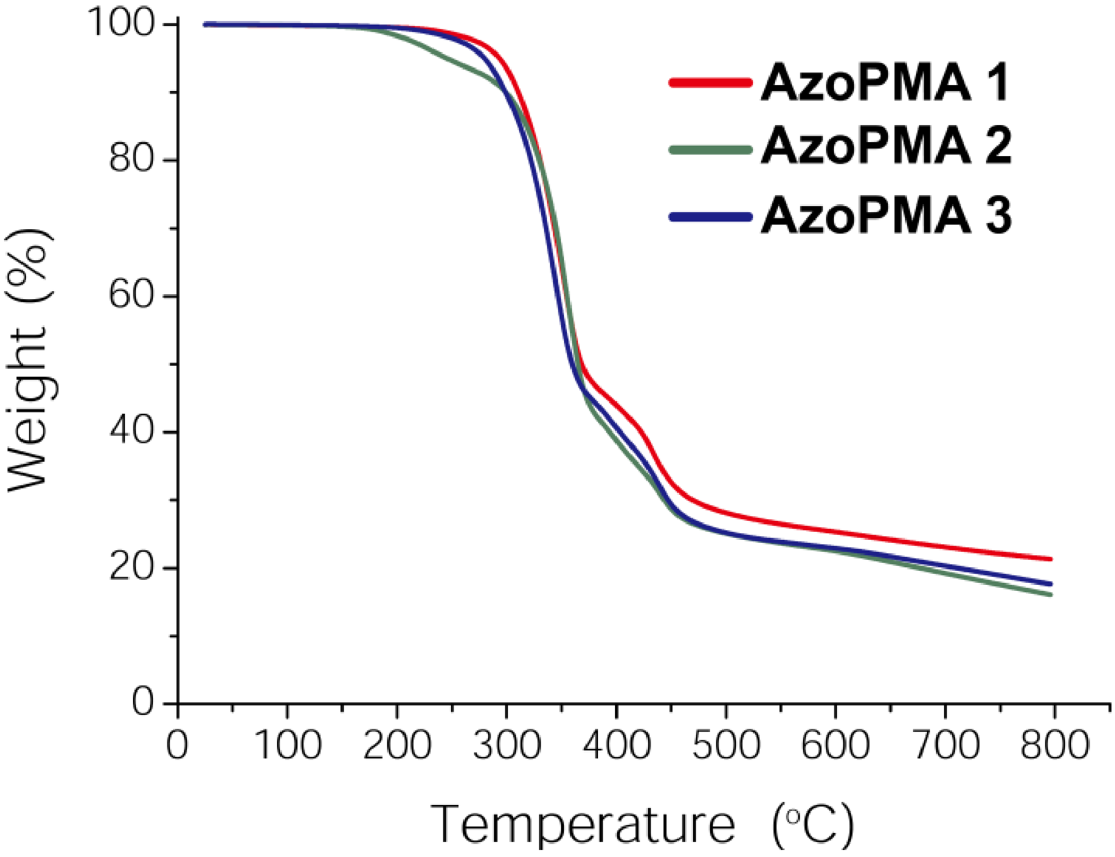

Supplementary Figure S20. TGA curves of AzoPMA **1**, **2**, and **3**.

S3. DSC curves of AzoPMAs.

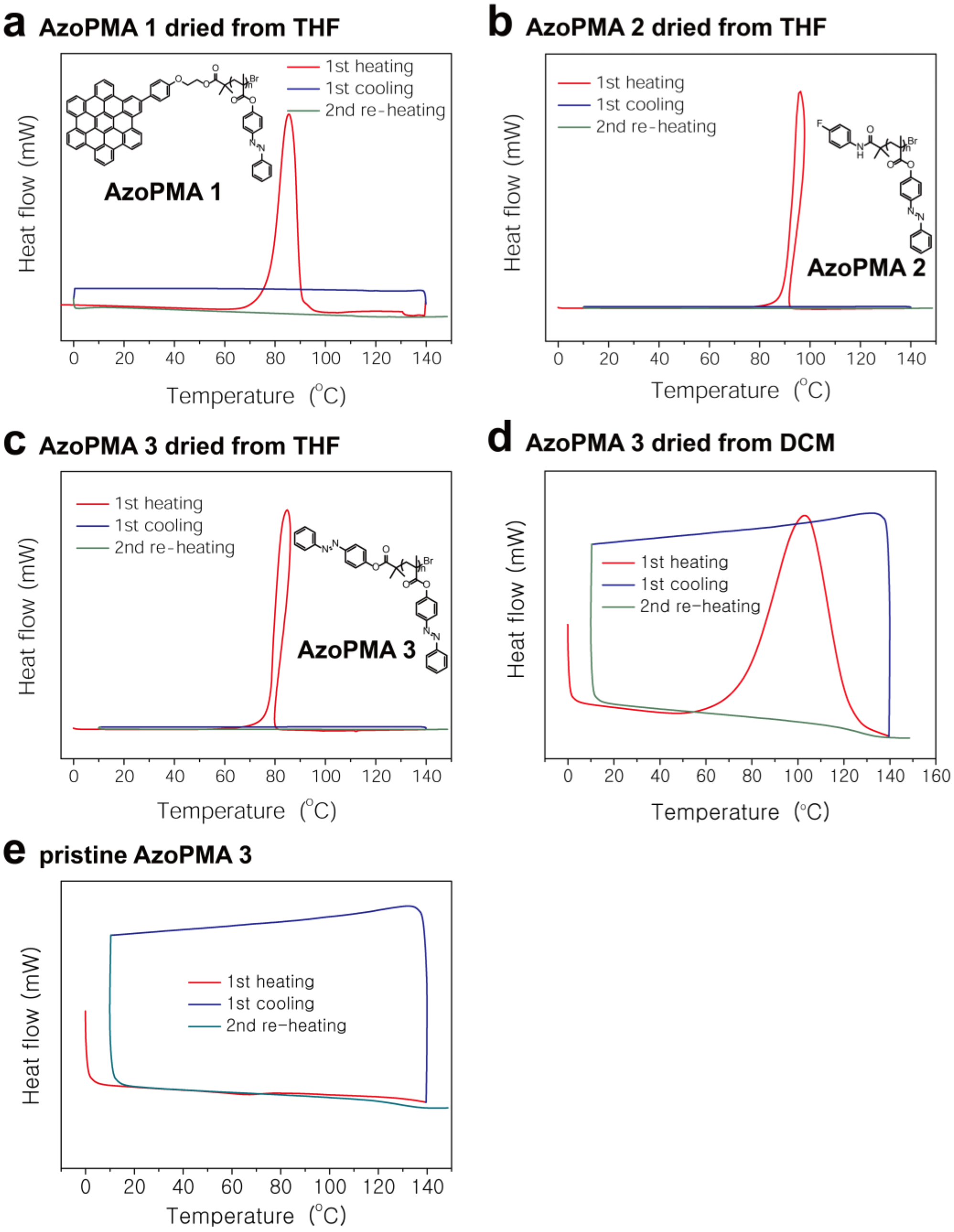

Supplementary Figure S21. DSC curves of AzoPMA polymers. (**a**) DSC curves of *Z*-AzoPMA **1** dried from THF. (**b**) DSC curves of *Z*-AzoPMA **2** dried from THF. (**c**) DSC curves of *Z*-AzoPMA **3** dried from THF. (**d**) DSC curves of *Z*-AzoPMA **3** dried from DCM. (**e**) DSC curves of pristine AzoPMA **3** (*E*-isomer) dried from THF.

S4. Non-monotonic character of the temperature during the DSC measurement

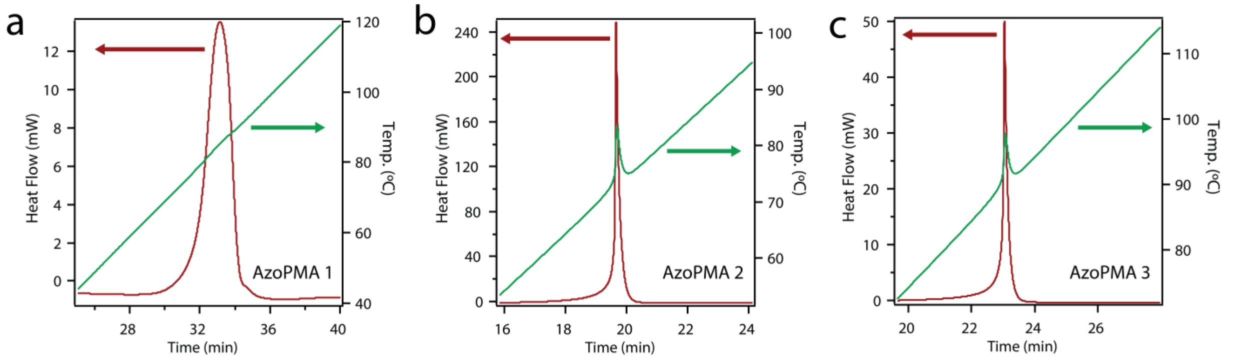

Supplementary Figure S22. Heat flow and Temp. vs time during DSC measurements of (**a**) AzoPMA **1**. (**b**) AzoPMA **2**. (**c**) AzoPMA **3**.

S5. DSC curve of AzoPMA 3

S5.1. THF photo-degradation products test

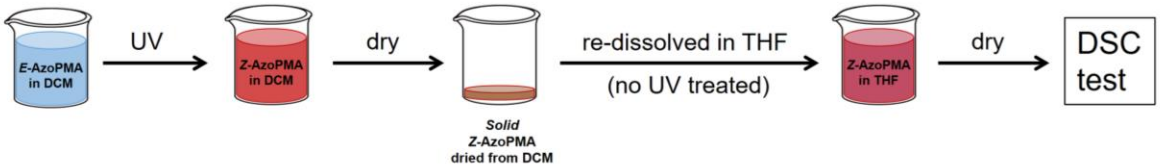

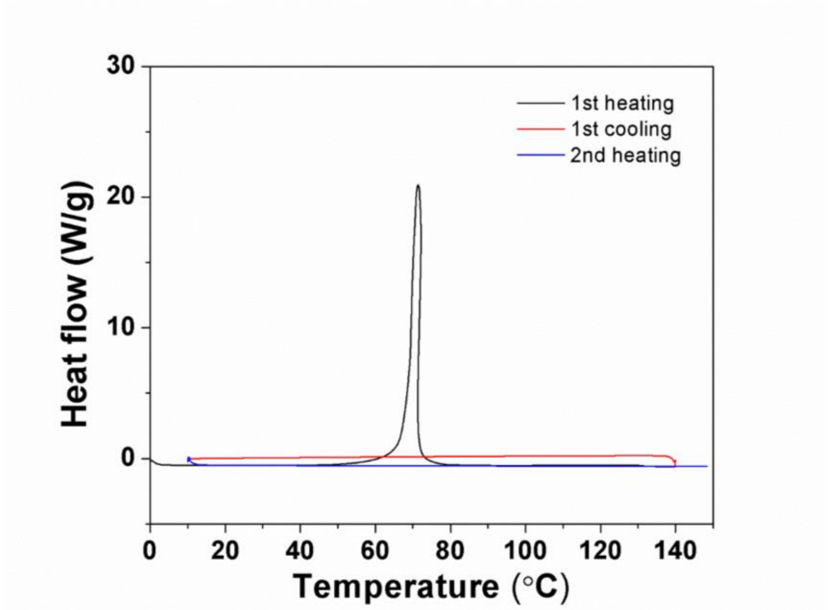

Supplementary Figure S23. DSC curves of AzoPMA **3** fabricated from the sample preparation method above.

S5.2. THF impurity test

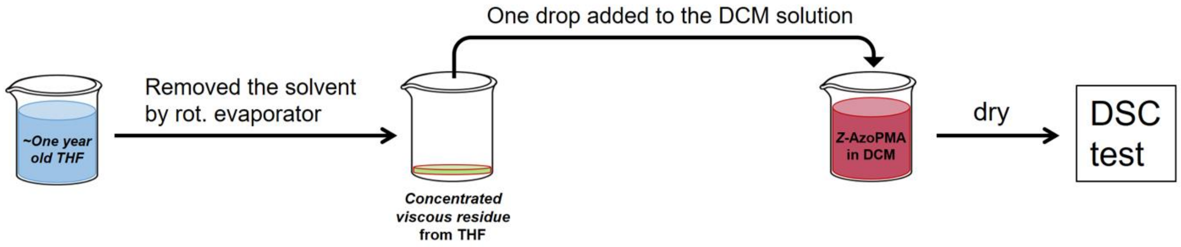

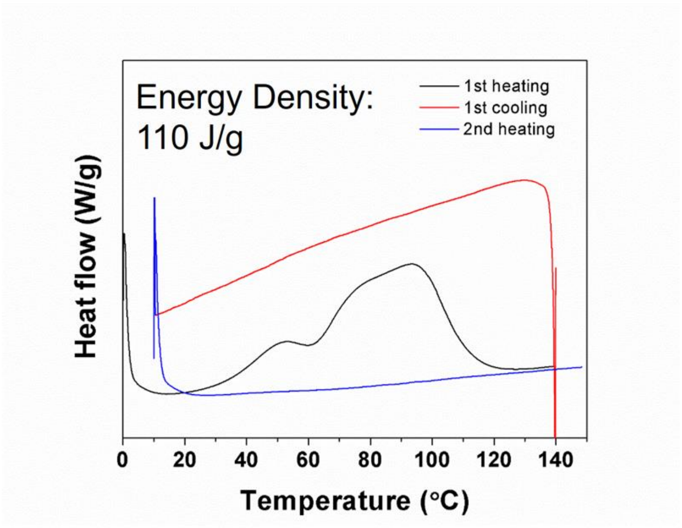

Supplementary Figure S24. DSC curves of AzoPMA **3** fabricated from the sample preparation method above.

S6. Elemental analysis of AzoPMA 3 by EDX

AzoPMA **3** films on Si substrate were used to check copper residue by EDX.

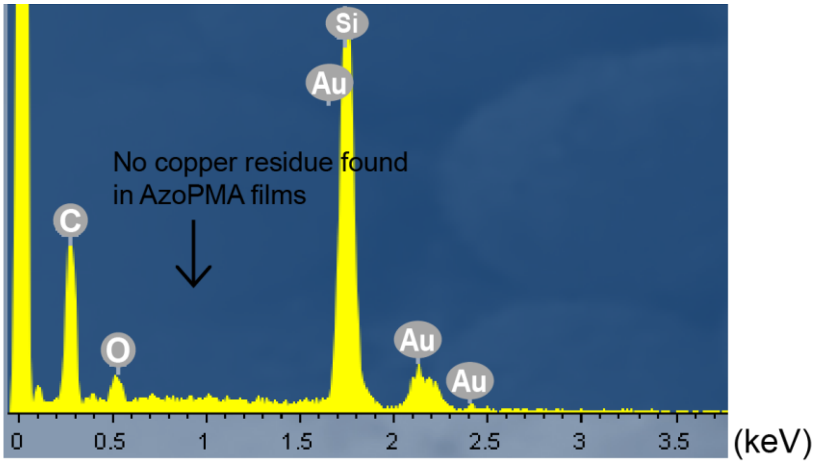

Supplementary Figure S25. EDX image of solid AzoPMA **3** films dried from DCM.

S7. Solution-State Isomerization Kinetics

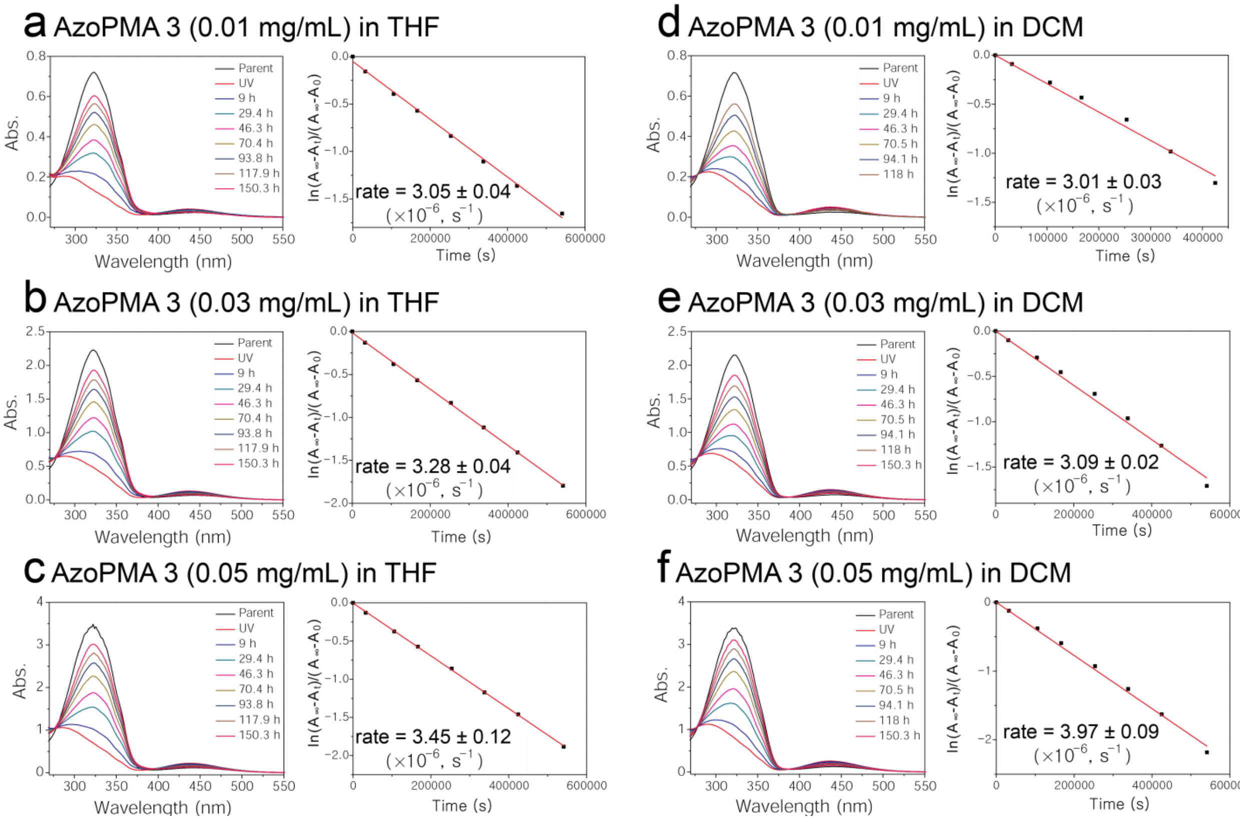

Supplementary Figure S26. UV-vis absorption spectra and rate of AzoPMA **3** in THF or DCM at the different concentrations.

S8. Glass temperature (T_g) of AzoPMA

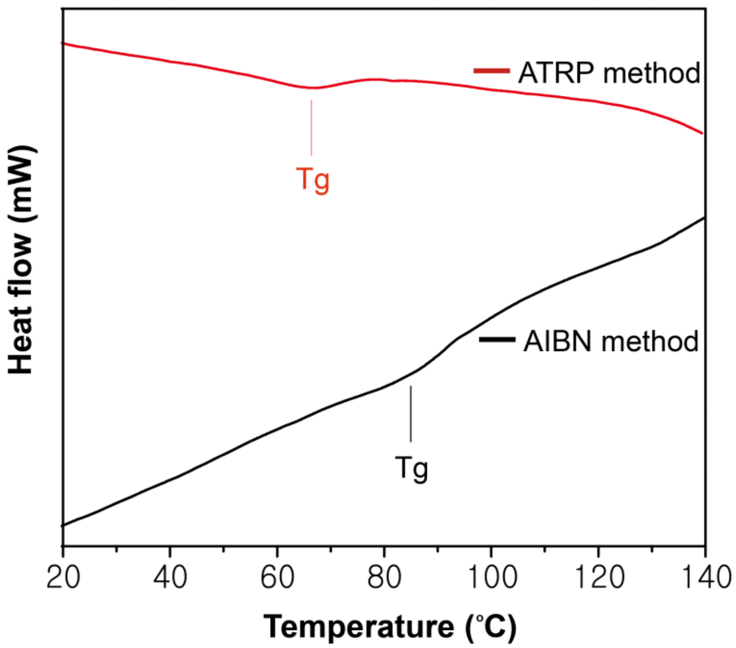

Supplementary Figure S27. 1st heating DSC curve of pristine AzoPMA **3** polymerized from controlled radical polymerization method (red) and pristine AzoPMA **4** polymerized from free radical polymerization method (black).

S9. Solvent effect of DCM vs THF

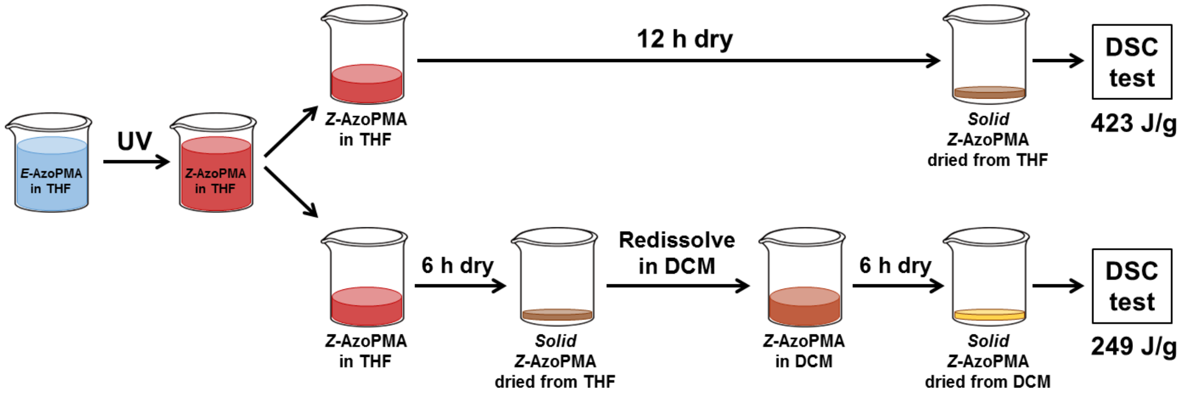

Supplementary Figure S28. Scheme of checking solvent effect of DCM vs THF sample.

S10. Glass temperature (T_g) of AzoPMA after heat release.

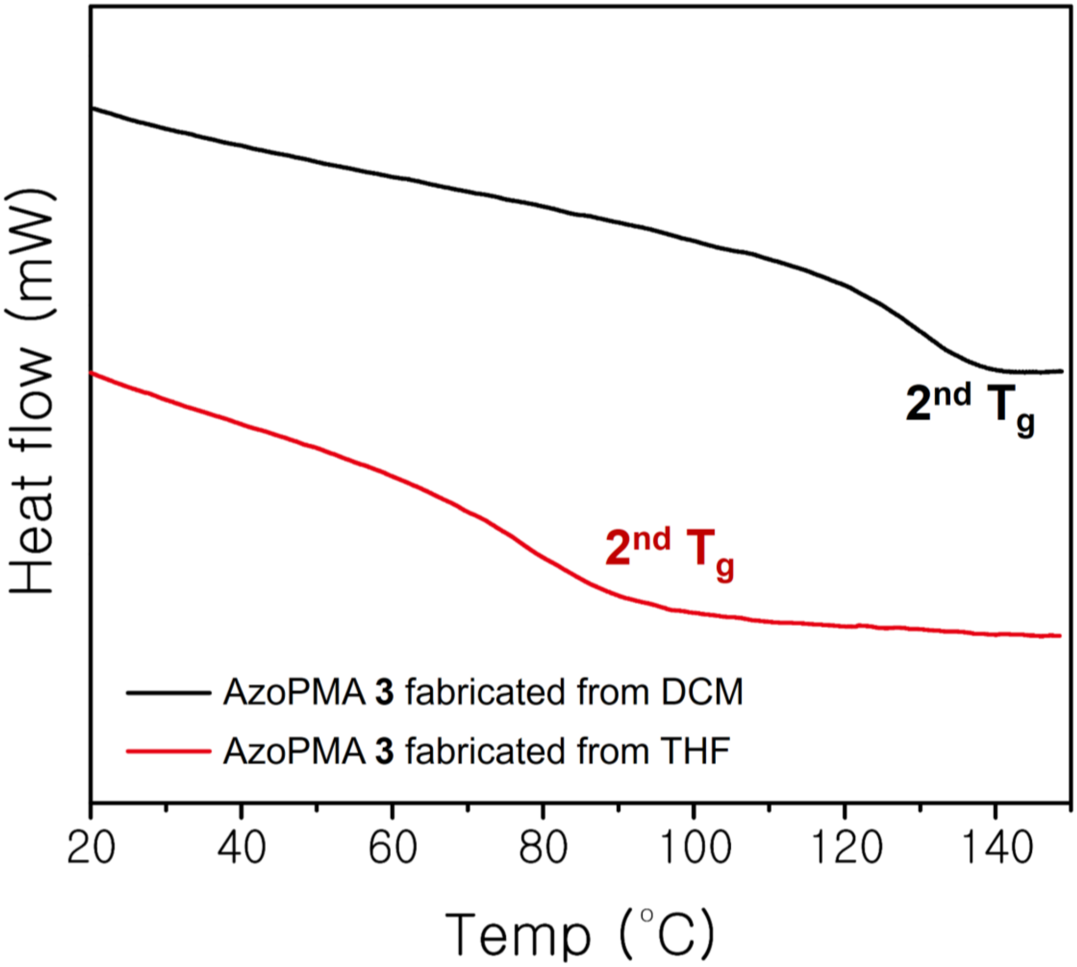

Supplementary Figure S29. T_g of AzoPMA obtained from DCM and THF sample after heat release (2nd heating DSC curve after exotherm).

S11. Stability test of AzoPMA 3 by NMR.

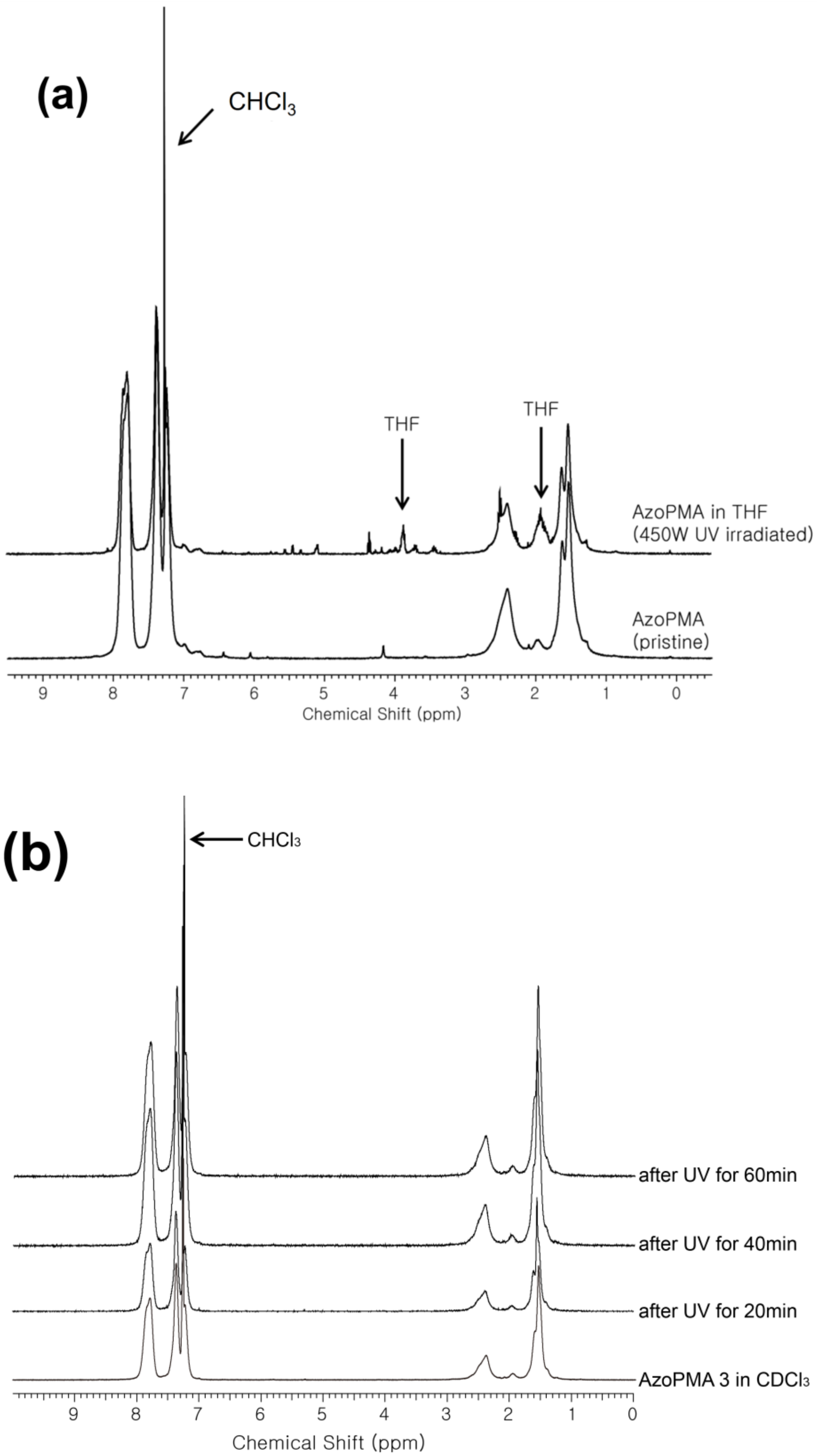

Supplementary Figure S30. ^{1}H NMR ($CDCl_3$) of (a) UV irradiated AzoPMA vs pristine AzoPMA, and (b) UV irradiated AzoPMA in $CDCl_3$.

S12. Surface morphology of AzoPMA by SEM

To prepare SEM samples, the Si surface was cleaned by piranha solution for 30 min. Solid AzoPMA **3** films on the Si substrate are prepared by drop casting of AzoPMA **3** solution (1 mg/mL) in DCM or THF, respectively. After drying for 1 h, the AzoPMA **3** films were coated by Au sputtering for 60 s. (SEM operation condition: 5 kV, 100 pA)

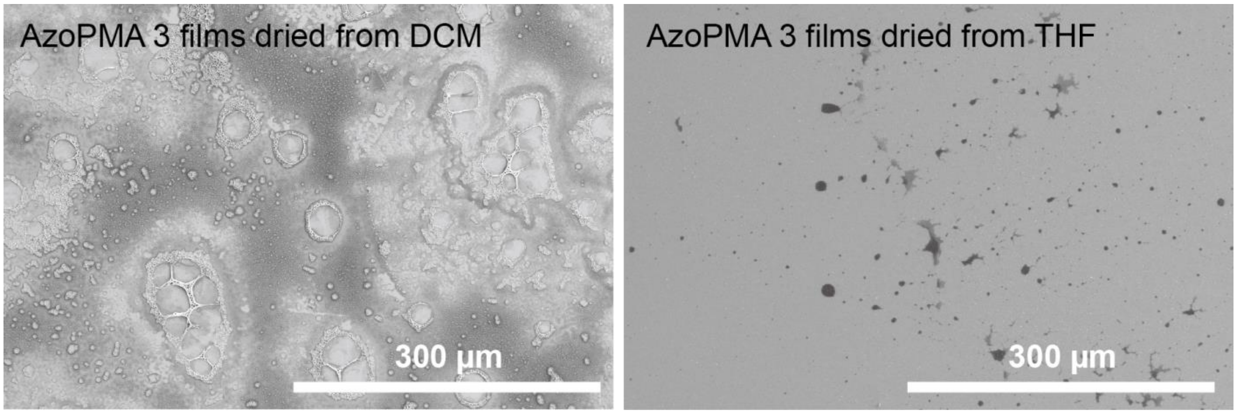

Supplementary Figure S31. SEM image of solid AzoPMA **3** films dried from DCM vs THF.

S13. UV charging condition: solution vs solid state

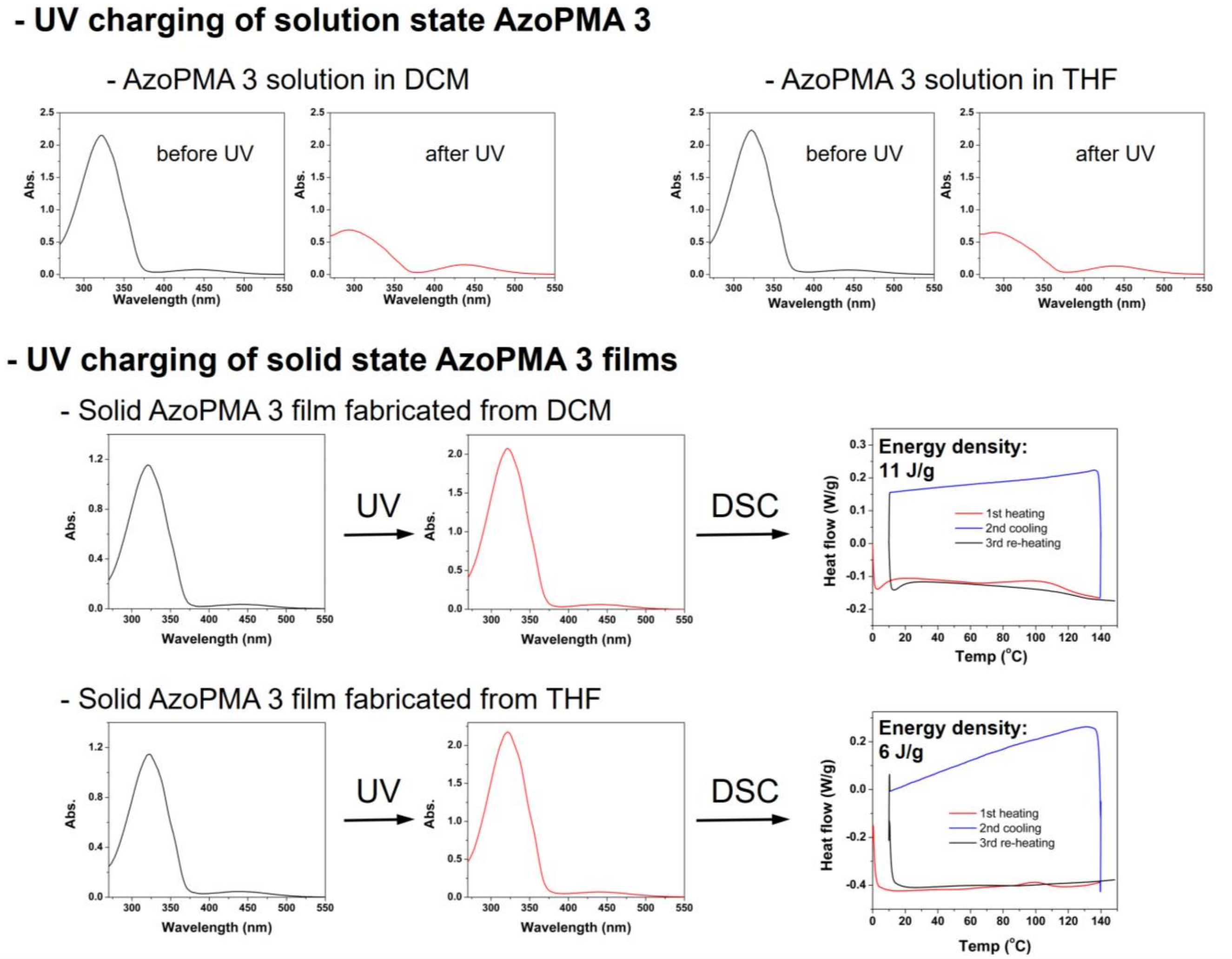

Supplementary Figure S32. UV-Vis and DSC curve after UV radiation in solution vs solid state AzoPMA **3**.

S14. Repeatability

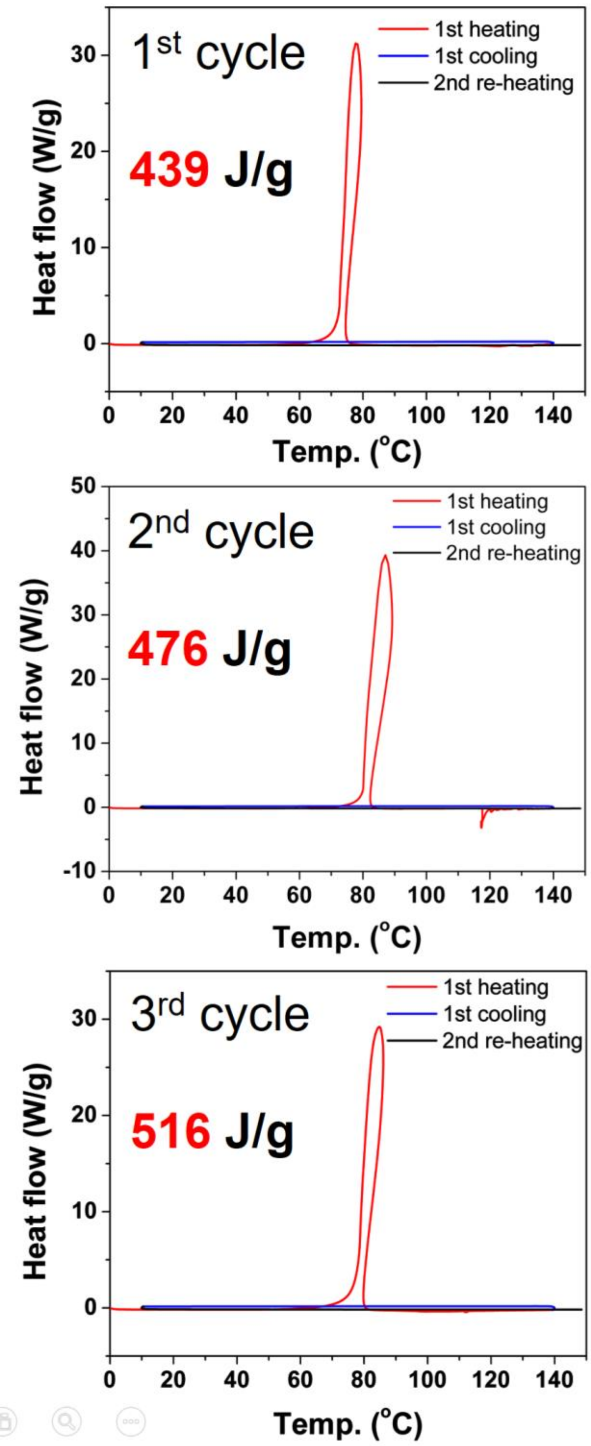

Supplementary Figure S33. Repeatability test of AzoPMA **2**.

S15. Polymerization Method comparison

Recently, Zhitomirsky *et al.*[4] reported that an energy density of ~110 J/g for azobenzene anchored on PMA-based polymer similar to AzoPMA 3 that is substantial lower than the values we observed. Moreover, their reported exotherms were broad compared to ours reported in this manuscript[4]. We identified two major differences between our work and the reported work of Zhitomirsky *et al.*: (1) polymerization method and (2) solvent processing for fabricating films.

First, they used uncontrolled radical polymerization using AIBN as the initiator. We used SARA ATRP, a controlled radical polymerization method. From ^{13}C NMR of AzoPMA 3, we concluded that AzoPMA **3** (M_n: 14,811, M_w: 25,745, *Đ*: 1.73) is syndiotactic rich with 73.6 % of rr, 22.9 % of mr, and 3.5 % of mm (See Supplementary Fig. S15). We then synthesized AzoPMA (AzoPMA 4, M_n: 12,842, M_w: 21,446, *Đ*: 1.67, see Supplementary Fig. S16-18) using free radical polymerization using AIBN as the initiator, similar to the method used by Zhitomirsky *et al.*. ^{13}C NMR of this polymer showed that it also had rich syndiotacticity with 74.7 % of rr, 23.0 % of mr, and 2.3 % of mm (See Supplementary Fig. S19). Based on these observations, we concluded that both polymerization methods provided a highly syndiotactic polymer. However, the two polymers do have different glass transition temperatures (T_g). We measured T_g = 62 °C for controlled radical polymerization and T_g = 89 °C for free radical polymerization. This result indicates that each polymer structural configuration and polymer neighboring environment are different, even though the estimated syndiotacticity from ^{13}C NMR are similar (see Supplementary Fig. S27).

Second, Zhitomirsky *et al.*[4] fabricated solid AzoPMA films from toluene, charged it under UV, redissolved it into DCM (or toluene), re-dried it on the DSC pan, and then used DSC to evaluate the energy density. We used THF or DCM solvent for charging and fabricating films. Zhitomirsky *et al.* claimed that the solvent had no effect on the energy density of AzoPMA system, which is inconsistent with our observations. To further confirm our observations, we charged AzoPMA **3** in THF and then

split into two parts (see Supplementary Fig. S28). The solvent from the first part was removed *in vaccuo*, and then further dried in the vacuum oven for 12 h at ambient temperature. DSC indicated that the energy density of this sample was 423 J/g. The solvent from the second part was removed the solvent *in vaccuo*, further dried in vacuum oven for 6 h, and re-dissolved in DCM. The DCM solvent was removed again under reduced pressure and the sample was dried for 6 h under ambient conditions. This sample showed an energy density of ~249 J/g. These results show again that the energy density is affected by the solvent that is used to prepare solid samples. These results are consistent with our conclusion that the solvent used to process the polymer must not interact too strongly with the backbone such that it may solvate the charged *Z* state. If the *Z* isomer is not sufficiently solvated in solution, we observe aggregation, which leads to films with misaligned dipoles, resulting in a loss of any cooperative benefits that give high-energy density solids.

Reference

1 **Jeong, S. P., Boyle, C. J. & Venkataraman, D. Poly(methyl methacrylate) end-functionalized with hexabenzocoronene as an effective dispersant for multi-walled carbon nanotubesl. *RSC Adv.* 6, 6107-6110, doi:10.1039/c5ra19883f (2016).**

2 **Lu, Y. Y. & Moore, J. S. Semi-fused hexaphenyl hexa-peri-hexabenzocoronene: a novel fluorophore from an intramolecular Scholl reaction. *Tetrahedron Lett.* 50, 4071-4077, doi:10.1016/j.tetlet.2009.04.103 (2009).**

3 **Zhang, D. P. *et al.* A dumbbell-like supramolecular triblock copolymer and its self-assembly of light-responsive vesicles. *RSC Advances* 5, 47762-47765, doi:10.1039/c5ra08661b (2015).**

4 **Zhitomirsky, D., Cho, E. & Grossman, J. C. Solid-State Solar Thermal Fuels for Heat Release Applications. *Adv. Energy Mater.* 6, 1502006, doi:10.1002/Aenm.201502006 (2016).**
